# Decentralized Mesh-Based Model Predictive Control for Swarms of UAVs

**DOI:** 10.3390/s20154324

**Published:** 2020-08-03

**Authors:** Salvatore Rosario Bassolillo, Egidio D’Amato, Immacolata Notaro, Luciano Blasi, Massimiliano Mattei

**Affiliations:** 1 Department of Engineering, University of Campania Luigi Vanvitelli, 81031 Aversa (CE), Italy; salvatorerosario.bassolillo@unicampania.it (S.R.B.); immacolata.notaro@unicampania.it (I.N.); luciano.blasi@unicampania.it (L.B.); massimiliano.mattei@unicampania.it (M.M.); 2 Department of Science and Technology, University of Naples Parthenope, 80143 Naples, Italy

**Keywords:** UAV swarm, formation flight, decentralized control, anti-collision, obstacle avoidance

## Abstract

This paper deals with the design of a decentralized guidance and control strategy for a swarm of unmanned aerial vehicles (UAVs), with the objective of maintaining a given connection topology with assigned mutual distances while flying to a target area. In the absence of obstacles, the assigned topology, based on an extended Delaunay triangulation concept, implements regular and connected formation shapes. In the presence of obstacles, this technique is combined with a model predictive control (MPC) that allows forming independent sub-swarms optimizing the formation spreading to avoid obstacles and collisions between neighboring vehicles. A custom numerical simulator was developed in a Matlab/Simulink environment to prove the effectiveness of the proposed guidance and control scheme in several 2D operational scenarios with obstacles of different sizes and increasing number of aircraft.

## 1. Introduction

Research on UAV has attracted increasing attention for at least two decades, exploring several topics, such as design, development and deployment. Military and civil applications are exponentially growing thanks to the great versatility and adaptability of these kind of platforms to a great number of missions. Furthermore, costs are decreasing more and more due to the technological developments driven by a greater attention and use.

However, it is not possible for a mission to always be completed effectively by a single UAV, as it may require the cooperation of several UAVs to perform complex tasks. This need may also derive from the choice to use smaller and lighter vehicles to reduce costs and increase reliability. In fact, costs of a single aircraft exponentially increase with the take-off weight [1,2,3,4], whereas the use of several smaller UAVs can also be effective in reducing the production and even operating costs [5,6]. The reduction of the mission time can be another important reason for the use of more than one aircraft whenever the limited endurance of a single UAV forces it to take off and land several times for refueling. Furthermore, the robustness of the overall mission can be enhanced by creating redundancy and reducing the payload weight per aircraft [7,8,9]. Finally, by involving several smaller vehicles having an improved mobility in confined spaces, it is possible to survey larger areas with respect to a bigger single aircraft, even if equipped with higher-quality sensors.

In the literature, several examples can be found regarding swarms of UAVs and related applications. An extended research field focuses on the swarming behavior, called *flocking*, known in nature as the collective behavior of a large number of agents able to interact and share a common objective [10,11,12,13,14,15,16,17,18,19]. Flocking techniques deal with the ability of a group of autonomous agents (being aircraft or robots) to move like a swarm of birds or insects.

Tracking a static or moving target has been studied in [20], where multiple UAVs searched for a source of pollution while keeping a safe distance from each other. An example of group of aircraft looking for targets using random walk paths can be found in [21], where an ant colony-like heuristic algorithm was considered. By depositing a virtual pheromone, the probability that other aircraft pass the same path was increased. Cooperative surveillance is another investigated task involving a swarm of UAVs: in [22], an evolutionary-based optimization technique was used to find the optimal position of UAVs in the airspace that maximizes the coverage of the area of interest, whereas in [23] a distributed algorithm was considered, taking into account communication constraints. In [24], a cognitive adaptive optimization algorithm was presented, having the goal of maximizing the surveilled area with a team of aircraft. In [25], a behavior-based algorithm leading a swarm of UAVs during a surveillance mission was presented.

However, the key point for an effective use of a swarm is the capability of the aircraft to properly collaborate in order to accomplish the mission objectives by coordinating their tasks.

An example of a collaboration concept consists of constrained flight between multiple UAVs, as in the case of several drones that work linked together to transport heavy loads [26,27,28,29,30].

Another example of a collaboration concept is the use of fleets of fixed-wing aircraft to improve the aerodynamic efficiency during flight [31]. This concept falls into the more general problem of managing the aircrafts’ relative positions to optimize performance in terms of fuel consumption or time spent to achieve mission goals [32,33,34,35,36,37].

Generally speaking, the output of a swarm control algorithm consists in the computation of the appropriate control signals to maintain the shape of the swarm, satisfying the assigned performance constraints [38]. For example, flocking research focuses on consensus-based formation control [15], which uses the relative position of the vehicles to realize a certain shape of the flight formation. Formation control algorithms focus mainly on stability and performance constraints. The classic flocking control model is based on three heuristic rules [39]: cohesion, separation and alignment. The concept of using flexible swarm shapes has been developed only in recent years.

Another important research field is cooperative motion planning, which is based on collaboration among vehicles with different point of views. Taking into account mission goals (usually in terms of waypoints) and environmental constraints, such as obstacles or no-fly zones, aircraft are driven along optimal trajectories, while benefiting from coordination [40]. Cooperative motion planning algorithms mainly focus on safety distance from obstacles, computational time, swarm shape and trajectory smoothness [41,42].

To maintain the given shape of the flight formation, several control architectures have been proposed: leader–follower, virtual structure and behavior-based.

The leader–follower approach considers a leader working as a reference aircraft in the swarm with full information about the other vehicles. Some papers introduce the idea of a virtual leader in order to overcome the issues of centralization [43,44,45].

A virtual structure architecture was proposed in [46]. According to this approach, the vehicles keep a rigid geometric relationship to each other in order to maintain a reference shape [47]. This concept considers the formation shape as a rigid body, maintained by minimizing the position error between the virtual structure and the actual aircraft position.

Behavior-based formation control was first proposed in [48]. It deals with the formation control problem by using a hybrid vector-weighted control function, which is able to compute the command action based on several kinds of formation missions. Applications of this approach to large-scale robot formations can be found in [49,50,51,52].

Within the flocking research field, one of the most popular approaches to control flocking behavior is the use of artificial potential fields and consensus [38,53,54]. Flock aggregation and collision avoidance are often dealt with using a potential field, whose design is still an active field of research [55].

Another common approach is based on the use of the nonlinear dynamic inversion technique (NDI). Through the use of nonlinear functions, the NDI control technique attempts to cancel out the inherent dynamics of a plant and enforce the dynamics of a reference model. The most important benefit is the ability to linearize systems. The resulting model can be controlled by any technique, such as sliding model control (SMC) [56,57] and pole placement [58,59], $H_{\infty }$ [60]. The authors of [61] dealt with a decentralized algorithm for a swarm of UAVs that uses the potential field approach combined with a sliding mode control technique. Optimal control is another option that can be used to compute the control command to achieve swarm objectives while preserving performance and environmental constraints. A stochastic optimal control approach was presented in [62], whereas linear and nonlinear MPCs. were presented in [63,64,65].

In several papers, authors used a machine learning heuristics to tackle the problem of controlling a swarm. For example, [66] described a task assignment algorithm using a self-reinforcement learning model to specialize the vehicles for the different task types, balancing the work load; another example is the use of the ant colony algorithm to organize task assignment: in [36] a swarming technique was developed to perform distributed target recognition using UAVs. In [67], multiple UAVs were involved in looking for targets that are displaying evading behavior in order to avoid detection. In [68], a genetic algorithm was used to optimize the tuning of the consensus control parameters of a swarm of quadrotors. In this work, UAVs try to find an efficient geometric flight configuration in order to optimize their sensing capabilities. Nature-inspired optimization algorithms were also used in reconfiguration problems [69,70].

Scalability and decentralization represent two other important issues in swarm management strategy [61]. They are strictly related to the swarm architecture and control approach used to maintain the formation. While several leader–follower architectures are centralized on the leader, computing the desired positions for each aircraft, flocking techniques are usually decentralized, in order to mimic the natural behavior.

In this paper, the design of a decentralized flocking strategy is dealt with for a fleet of UAVs flying at a given altitude to reach a target position while maintaining an assigned connection topology with desired mutual distances.

The starting point of the proposed approach is the triangle formation algorithm (TFA), previously described in [71,72]. Such a technique represents an interactive control algorithm used to organize the swarm into an equilateral triangle-based formation. However, TFA does not assign a precise topology to the formation and may have some issues in the presence of obstacles, allowing the formation of sub-swarms that may not rendezvous with the main formation.

This is an important open question concerning the spreading of the formation in the presence of obstacles. Several approaches use a virtual leader, allowing the followers to spread between obstacles without creating sub-swarms with a prescribed topology [73,74,75].

In this paper, we propose a new strategy, based on an extension of the Delaunay triangulation algorithm: in the absence of obstacles, the assigned topology guarantees a single regular-meshed flight formation, whereas in the presence of obstacles, it allows the formation to split into independent sub-formations to avoid collisions, forcing them to rendezvous after obstacles.

The application of the Delaunay triangulation in the presence of obstacles represents a novel aspect requiring the definition of a visibility property. Furthermore, while the idea of triangular mesh-based formation has already been exploited [72], the new feature consists in the capability to generate sub-swarms without the definition of any physical or virtual leader.

Another original point concerns trajectory tracking and collision avoidance: TFA computes the desired position of the aircraft to accomplish the triangle-based formation. This is tracked by a proportional navigation-based control algorithm that cannot take into account performance and environmental constraints. In [72], an artificial potential field was also used to take obstacles into account. Our approach is to use a potential field [76,77] to compute the aircraft’s desired heading angle and speed to keep the desired mutual distances between aircraft, whereas an MPC-based control algorithm performs the trajectory tracking and the obstacle collision avoidance.

In our previous paper [78], we presented an MPC strategy in the absence of obstacles with prescribed rules between aircraft. Based on this work, we propose an original reactive MPC aimed at improving the performance of the anti-collision scheme using practical geometrical constraints leading to a computationally tractable algorithm.

The paper is organized as follows:

- In Section 2, the problem is precisely stated and the general scheme of the proposed strategy is illustrated;
- Section 3 focuses on the swarm topology based on the Delaunay triangulation;
- Section 4 describes the potential based guidance algorithm driving the swarm on a regular-meshed flight formation;
- Section 5 focuses on the MPC-based trajectory tracking algorithm with collision avoidance;
- Finally, in Section 6, numerical simulation results are shown for several operational scenarios.

## 2. Problem Statement and General Architecture

Consider a swarm of *N* vehicles flying at fixed speed and altitude to simulate a cruise or surveillance mission phase.

As the focus of this paper is mainly on swarm guidance and collision avoidance, we neglected any atmospheric disturbance as well as any change in aircraft properties (i.e., mass variation due to fuel consumption).

We then assume that each aircraft is equipped with its own flight control system (FCS), so the *i*-th vehicle’s closed loop dynamics can be described well by the following simple kinematic equations:

(1) $$\left\{\begin{array}{ccc} {\dot{x}}_{i}\left(t\right) & = & V_{i}\left(t\right)\cos\psi_{i}\left(t\right)\\ {\dot{y}}_{i}\left(t\right) & = & V_{i}\left(t\right)\sin\psi_{i}\left(t\right)\\ {\dot{V}}_{i}\left(t\right) & = & u_{V_{i}}\left(t\right)\\ {\dot{\psi }}_{i}\left(t\right) & = & u_{\psi_{i}}\left(t\right) \end{array}\right.$$

where $P_{i}\left(t\right)={\left[x_{i}\left(t\right),y_{i}\left(t\right)\right]}^{T}$ is the position at time *t*, $V_{i}\left(t\right)$ is the speed, $\psi_{i}\left(t\right)$ is the heading angle $u_{\psi_{i}}\left(t\right)$ and $u_{V_{i}}\left(t\right)$ are the input control signals in terms of turning rate and acceleration along the flight trajectory, respectively.

The goal of the fleet is to reach a given target position, namely $P^{f}={\left[x^{f},y^{f}\right]}^{T}$, preserving the mutual distance between pairs of aircraft.

**Figure 1:**
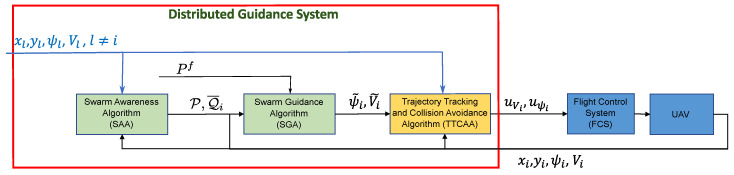
On-board guidance and control algorithm architecture (time information has been omitted).

Figure 1, shows the architecture of the guidance algorithm implemented on each aircraft. This is based on the following components:

- The so-called swarm awareness algorithm (SAA) aims to compute the connection topology ($P,{\bar{Q}}_{i}$) using an extended Delaunay triangulation technique that takes into account the presence of obstacles.
- The swarm guidance algorithm (SGA) is based on a potential field technique to compute the desired heading angle and speed (${\tilde{\psi }}_{i},{\tilde{V}}_{i}$) so as to maintain the mutual distances between aircraft and reach the target position $P^{f}$.
- The trajectory tracking and collision avoidance algorithm (TTCAA) is based on a model predictive controller and provides the reference signals $u_{\psi_{i}}$ and $u_{V_{i}}$ to the FCS, taking into account environmental constraints (obstacles, no-fly zones, other aircraft) and possible constraint on aircraft state and input (e.g., minimum/maximum speed, maximum turn rate).

In order to take into account a digital implementation, the algorithm works with a fixed sampling period Δt. In the following the discrete time $t_{k}=t_{k-1}+\Delta t$ is referred to as *k*.

The proposed strategy is based on the assumption that each vehicle can measure or estimate its current position, speed and heading. Moreover, it is equipped with an ADS-B or a similar device to share these estimates with the other vehicles.

## 3. Swarm Awareness Algorithm

The SAA is based on the Delaunay triangulation [79] with the addition of a visibility concept.

**Definition** **1.** *Given the set $P\left(k\right)=\{P_{l}\left(k\right),\;l=1,2,\cdots ,N\}$ of positions of the swarm vehicles, the Delaunay triangulation, $D\left(P\left(k\right)\right)=\left\{T_{jml}:\forall \;P_{j}\left(k\right),P_{m}\left(k\right),P_{l}\left(k\right)\in P\left(k\right)\right\}$, is a triangular partition of the swarm such that no position $P_{i}\left(k\right)\in P\left(k\right)$ is inside the circumcircle of any triangle $T_{jml}\left(k\right)\in D\left(P\right)$, (j,m,l≠i).*

**Definition** **2.** *Given a Delaunay triangulation, the associated Delaunay triangulation graph (DTG) G(k)={V(k),A(k)} is composed of the nodes set V(k)=P(k) and the arc set A(k) containing all the pairs $<P_{m}\left(k\right)P_{l}\left(k\right)>$ such that the segment $\bar{P_{m},P_{l}}$ is an edge of a Delaunay triangle.*

**Definition** **3.** *A vehicle m is a neighbor of the vehicle l, at time k, if the pair $<P_{m}\left(k\right),P_{l}\left(k\right)>\in A\left(k\right)$. The set of neighboring aircraft of the j-th vehicle is:*

(2) $$Q_{j}\left(k\right)=\left\{m:\;<P_{m}\left(k\right),P_{j}\left(k\right)>\in A\left(k\right)\right\}$$

In the presence of obstacles, which are modeled as flat geometrical figures, in order to implement an obstacle-avoidance strategy based on the possible splitting of the swarm into independent sub-swarms, a visibility concept is also introduced.

**Definition** **4.** *At time k, aircraft j is visible from aircraft i if segment $\bar{P_{i}\left(k\right)P_{j}\left(k\right)}$ does not intersect any obstacle. The set of visible neighbors of the aircraft i is*

(3) $${\bar{Q}}_{i}\left(k\right)=\left\{j\in Q_{i}:\;\Phi_{ij}\left(k\right)=1\right\}$$

*where $\Phi_{ij}\left(k\right)$ is the visibility operator*

(4) $$\Phi_{ij}\left(k\right)=\Phi_{ji}\left(k\right)=\left\{\begin{array}{c} 1\;\mathrm{if}\;i\;\mathrm{is}\;\mathrm{visible}\;\mathrm{from}\;j\\ 0\;\mathrm{otherwise}. \end{array}\right.$$

According to the visibility concept, in the presence of obstacles, the DTG is modified by considering only arcs present in ${⋃}_{i=1}^{N}{\bar{Q}}_{i}\left(k\right)$. For this reason, DTG may become not connected and several subgraphs without common nodes may arise. However, once the obstacles have been overcome, the DTG becomes connected again, resulting in a rendezvous of the formation.

On each vehicle *i*, the SAA calculates the DTG and ${\bar{Q}}_{i}$ at each time step *k*.

## 4. The Swarm Guidance Algorithm

The SGA computes the guidance law to guarantee a correct position of the aircraft in the swarm while flying toward the target point. It is based on a local interaction between visible neighboring aircraft and a potential field approach [80,81] forcing the swarm vehicles to take place on the nodes of a regular triangular mesh with a given mutual distance $\bar{d}$.

At each discrete time step, the SGA computes a desired heading ${\tilde{\psi }}_{i}\left(k\right)$ and speed ${\tilde{V}}_{i}\left(k\right)$, which are then passed to the trajectory tracking algorithm described in the next section. In other terms, the SGA assures that the vehicle *i* moves toward a target direction $\Gamma_{i}\left(k\right)={\left[\cos{\tilde{\psi }}_{i}\left(k\right),\sin{\tilde{\psi }}_{i}\left(k\right)\right]}^{T}$ with a given speed.

**Figure 2:**
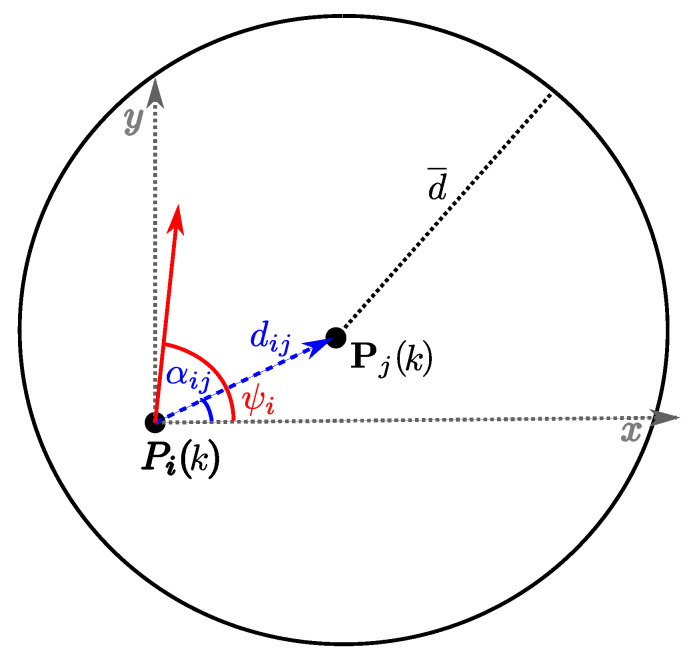
Reference angles for potential field computation. Red arrow represents the *i*-th vehicle’s flight direction. The repulsive force driven by *j* forces vehicle *i* to stay on the circumference with a radius equal to the desired distance $\bar{d}$.

As shown in Figure 2, let $d_{i,j}\left(k\right)=\left|\right|P_{j}\left(k\right)-P_{i}\left(k\right)\left|\right|$ be the distance between two aircraft, *i* and *j*, visible to each other. An isotropic artificial potential field forces aircraft *i* to stay on a circumference with radius $\bar{d}$ centered on $P_{j}\left(k\right)$, for all visible aircraft *j*. To achieve this goal, the desired direction $\gamma_{\mathrm{ij}}\left(k\right)={\left[x_{\gamma_{ij}}\left(k\right),y_{\gamma_{ij}}\left(k\right)\right]}^{T}$ derives from the attractive–repulsive action defined as:

(5) $$\begin{array}{c} x_{\gamma_{ij}}\left(k\right)=\left\{\begin{array}{cc} k_{1}\cdot \left(\bar{d}/d_{i,j}\left(k\right)-1\right)\cdot \cos\alpha_{ij}\left(k\right)\; & \mathrm{if}\;d_{i,j}\left(k\right)\leq \bar{d}\\ k_{2}\cdot \left(d_{i,j}\left(k\right)/\bar{d}-1\right)\cdot \cos\left(\alpha_{ij}\left(k\right)+\pi \right)\; & \mathrm{if}\;d_{i,j}\left(k\right)>\bar{d} \end{array}\right.\\ y_{\gamma_{ij}}\left(k\right)=\left\{\begin{array}{cc} k_{1}\cdot \left(\bar{d}/d_{i,j}\left(k\right)-1\right)\cdot \sin\alpha_{ij}\left(k\right)\; & \mathrm{if}\;d_{i,j}\left(k\right)\leq \bar{d}\\ k_{2}\cdot \left(d_{i,j}\left(k\right)/\bar{d}-1\right)\cdot \sin\left(\alpha_{ij}\left(k\right)+\pi \right)\; & \mathrm{if}\;d_{i,j}\left(k\right)>\bar{d} \end{array}\right. \end{array}$$

where $k_{1}$ and $k_{2}$ are positive coefficients used to tune the repulsive/attractive component in the overall potential field.

In order to establish a hierarchy in the propagation of the potential field action, a *relative position* operator $\Xi_{ij}\left(k\right)$ is then defined establishing whether aircraft *j* is flying ahead ($\Xi_{ij}\left(k\right)=1$) or behind ($\Xi_{ij}\left(k\right)=0$) aircraft *i*, where:

(6) $$\Xi_{ij}\left(k\right)=\left\{\begin{array}{cc} 1 & \mathrm{if}\;|\Delta \alpha_{ij}\left(k\right)|\leq \pi /2\\ 0 & \mathrm{if}\;|\Delta \alpha_{ij}\left(k\right)|>\pi /2 \end{array}\right.$$

and

- $\Delta \alpha_{ij}\left(k\right)={\left\lfloor \alpha_{ij}\left(k\right)-\psi_{i}\left(k\right)\right\rfloor }_{-\pi }^{\pi }$,
- the operator ${\left\lfloor \cdot \right\rfloor }_{-\pi }^{\pi }$ wraps the angle in the interval [−π,π],
- $\alpha_{ij}\left(k\right)=\tan^{-1}{\left\lfloor \frac{y_{j}\left(k\right)-y_{i}\left(k\right)}{x_{j}\left(k\right)-x_{i}\left(k\right)}\right\rfloor }_{0}^{2\pi }$ is the angle between the vector $P_{j}\left(k\right)-P_{i}\left(k\right)$ and the horizontal axis, and
- $\psi_{i}\left(k\right)$ is the heading of the *i*-th aircraft.

The final desired direction $\Gamma_{i}\left(k\right)=\left[x_{\Gamma_{i}}\left(k\right),y_{\Gamma_{i}}\left(k\right)\right]{,}^{T}$ of the *i*-th aircraft is computed as a weighted averaged action from the visible neighboring aircraft, combined with a potential action driving to the target point:

(7) $$\Gamma_{i}\left(k\right)=\frac{\sum_{j\in {\bar{Q}}_{i}\left(k\right)}\Xi_{ij}\left(k\right)\gamma_{ij}\left(k\right)}{\mathrm{card}({\bar{Q}}_{i}\left(k\right))}+k_{Target}\frac{P^{f}-P_{i}\left(k\right)}{∥P^{f}-P_{i}\left(k\right)∥}$$

where $\mathrm{card}({\bar{Q}}_{i}\left(k\right))$ represents the cardinality of the set ${\bar{Q}}_{i}\left(k\right)$. The $k_{Target}$ positive constant is chosen to balance the attraction to the target with all other actions.

The resulting heading angle is finally computed as follows:

(8) $${\tilde{\psi }}_{i}\left(k\right)={\left\lfloor \tan^{-1}\left(\frac{y_{\Gamma_{i}}\left(k\right)}{x_{\Gamma_{i}}\left(k\right)}\right)\right\rfloor }_{0}^{2\pi }$$

Additionally, the desired speed ${\tilde{V}}_{i}\left(k\right)$ is a combination of the reference cruise speed $V_{c}$ and a repulsive or attractive action from neighboring vehicles, $k_{V}>0$ being a tuning parameter:

(9) $$\tilde{V_{i}}\left(k\right)=V_{c}+k_{V}\sum_{j\in {\bar{Q}}_{i}\left(k\right)}\left(2\Xi_{ij}\left(k\right)-1\right)\frac{d_{i,j}\left(k\right)-\bar{d}}{|d_{i,j}\left(k\right)-\bar{d}|}\left|\right|\gamma_{\mathrm{ij}}\left(k\right)\left|\right|$$

In this way, vehicle *i* is attracted to vehicle *j* if the mutual distance $d_{i,j}\left(k\right)>\bar{d}$ and vice versa.

## 5. Trajectory Tracking and Collision Avoidance Algorithm

**Figure 3:**
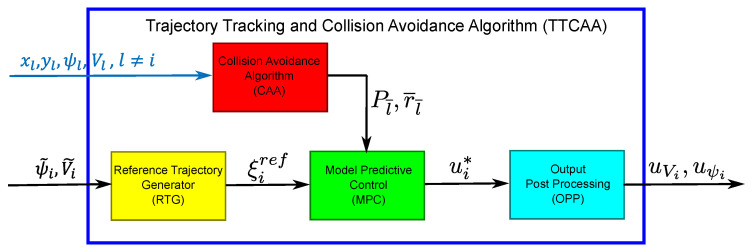
Trajectory Tracking and Collision Avoidance Algorithm.

The TTCAA runs on each aicraft *i* and is composed of the following parts (see Figure 3):

- A Reference Trajectory Generation (RTG): on the basis of the inputs from the SGA, it computes the reference trajectory for the MPC;
- A Collision Avoidance Algorithm (CAA): it adds constraints to the MPC problem, whenever a potential collision with other vehicles or obstacles is detected.
- Model Predictive Control (MPC): it computes the acceleration vector needed to follow the reference trajectory and avoid any potential collision;
- MPC Output Post Processing (OPP): it converts acceleration into control signal for the FCS, namely acceleration along the flight trajectory $u_{V_{i}}$ and turn rate $u_{\psi_{i}}$.

According to the MPC strategy, at each time step *k*, the algorithm computes the optimal control action $u_{i}^{*}\left(k|k\right)$ as the first element of the optimal sequence $U_{i}^{*}\left(k\right)={\left[u_{i}^{*}T\left(k|k\right),u_{i}^{*}T\left(k+1|k\right),\ldots ,u_{i}^{*}T\left(k+n_{c}|k\right)\right]}^{T}$, obtained by solving an optimal control problem over a finite prediction horizon $\left[k,k+n_{p}\right]$ [82].

Generally speaking the MPC requires the solution of the following problem.

**Problem** **1.** Constrained discrete time control optimization problem. Find the optimal sequence $U_{i}^{*}\left(k\right)$ composed of $n_{c}$ control moves, with $n_{c}\leq n_{p}$, such that:

(10) $$\begin{array}{c} U_{i}^{*}\left(k\right)=\underset{U_{i}}{\mathrm{argmin}}J\left(\xi_{i}\left(k|k\right),U_{i}\left(k\right),k\right) \end{array}$$

*subject to*

(11) $$\begin{array}{c} \eta \left(\xi_{i}\left(k+p|k\right),u_{i}\left(k+p|k\right),k\right)\leq 0\;\;\forall p\in \left[0,\ldots ,n_{p}\right] \end{array}$$

(12) $$\begin{array}{c} \xi_{i}\left(k+p+1|k\right)=f\left(\xi_{i}\left(k+p|k\right),u_{i}\left(k+p|k\right),k\right)\;\;\forall p\in \left[0,\ldots ,n_{p}\right]\\ \xi_{i}\left(k|k\right)=\xi_{i}\left(k\right) \end{array}$$

*where:*

- *J(·,·,·) is the cost function to be minimized;*
- *$\xi_{i}\left(k+p+1|k\right)$ is the predicted state at the time step $k+p+1,\forall p\in [0,\ldots ,n_{p}]$. This is computed using (12) which represents the dynamics of the aircraft, comprehensive of the initial condition ξ(k) which is measured or estimated;*
- *$u_{i}\left(k+p|k\right)$ is the control signal at the time instant $k+p,\forall p\in [0,\ldots ,n_{p}]$;*
- *Equation (11) defines static constraints involving states and inputs.*

In particular, to predict the aircraft state trajectory a linear kinematic model is considered, whose state-space representation is:

(13) $$\begin{array}{cc} \xi_{i}\left(k+1\right)= & \left[\begin{array}{cccc} 1 & 0 & \Delta t & 0\\ 0 & 1 & 0 & \Delta t\\ 0 & 0 & 1 & 0\\ 0 & 0 & 0 & 1 \end{array}\right]\xi_{i}\left(k\right)+\left[\begin{array}{cc} 0 & 0\\ 0 & 0\\ \Delta t & 0\\ 0 & \Delta t \end{array}\right]u_{i}\left(k\right)={A\xi }_{i}\left(k\right)+{\mathrm{Bu}}_{i}\left(k\right) \end{array}$$

where the state vector $\xi_{i}\left(k\right)={\left[x_{i}\left(k\right),y_{i}\left(k\right),v_{i,x}\left(k\right),v_{i,y}\left(k\right)\right]}^{T}$ is composed of the position $P_{i}\left(k\right)={\left[x_{i}\left(k\right),y_{i}\left(k\right)\right]}^{T}$ and the velocity vector $V_{i}\left(k\right)={\left[v_{i,x}\left(k\right),v_{i,y}\left(k\right)\right]}^{T}$ of the *i*-th aircraft, and $u_{i}\left(k\right)={\left[a_{i,x}\left(k\right),a_{i,y}\left(k\right)\right]}^{T}$ is the acceleration vector that must be optimized in order to allow the aircraft to follow the reference trajectory $\xi_{i}^{ref}\left(k\right)={\left[x_{i}^{ref}\left(k+p+1|k\right),y_{i}^{ref}\left(k+p+1|k\right),v_{i,x}^{ref}\left(k+p+1|k\right),v_{i,y}^{ref}\left(k+p+1|k\right)\right]}^{T}$ ($p=0,\ldots ,n_{p}$). This trajectory can be computed by integrating the following equation

(14) $$\left\{\begin{array}{c} x_{i}^{ref}\left(k+p+1|k\right)=x_{i}^{ref}\left(k+p|k\right)+v_{i,x}^{ref}\left(k+p|k\right)\Delta t\\ y_{i}^{ref}\left(k+p+1|k\right)=y_{i}^{ref}\left(k+p|k\right)+v_{i,y}^{ref}\left(k+p|k\right)\Delta t\\ v_{i,x}^{ref}\left(k+p+1|k\right)=\tilde{V_{i}}\left(k\right)\cos{\tilde{\psi }}_{i}\left(k\right)\\ v_{i,y}^{ref}\left(k+p+1|k\right)=\tilde{V_{i}}\left(k\right)\sin{\tilde{\psi }}_{i}\left(k\right) \end{array}\right.$$

over the prediction horizon $\left[k,k+n_{p}\right]$, starting from the initial condition $\xi_{i}^{ref}\left(k|k\right)={\left[x_{i}\left(k\right),y_{i}\left(k\right),{\tilde{V}}_{i}\left(k\right)\cos{\tilde{\psi }}_{i}\left(k\right),{\tilde{V}}_{i}\left(k\right)\sin{\tilde{\psi }}_{i}\left(k\right)\right]}^{T}$, where $\left[x_{i}\left(k\right),y_{i}\left(k\right)\right]$ represents the measured current position, ${\tilde{V}}_{i}\left(k\right)$ and ${\tilde{\psi }}_{i}\left(k\right)$ are provided by the SGA.

In order to obtain an online numerical solution to the MPC Problem 1 with Quadratic Programming (QP) techniques, the objective function is defined quadratic in the state and input variables as follows:

(15) $$\begin{array}{c} J\left(\xi_{i}\left(k|k\right),U_{i},k\right)=\sum_{p=0}^{n_{p}}e_{\xi_{i}}{\left(k+p+1|k\right)}^{T}Qe_{\xi_{i}}\left(k+p+1|k\right)+\\ +\sum_{p=0}^{n_{c}}u_{i}{\left(k+p|k\right)}^{T}Ru_{i}\left(k+p|k\right) \end{array}$$

The first term of the objective function *J* accounts for the difference between the predicted and reference trajectory, $e_{\xi_{i}}\left(\tau |k\right)=\xi_{i}\left(\tau |k\right)-\xi_{i}^{ref}\left(\tau |k\right)$, whereas the second one aims to limit the control effort. Weighting matrices Q and R are positive definite.

On the other hand, an effort has to be spent to formulate constraints as linear functions of the optimization variable.

### 5.1. Acceleration and Speed Limits

Control laws must be consistent with the aircraft acceleration and speed limits.

Speed is limited by the following nonlinear constraints:

(16) $$\begin{array}{c} V_{i,min}\leq V_{i}\left(k+p+1|k\right)\leq V_{i,max}\;\forall p=0,\ldots ,n_{p} \end{array}$$

where $V_{i,min}$ and $V_{i,max}$ are the minimum and maximum allowed speed and $V_{i}=\sqrt{v_{i,x}^{2}+v_{i,y}^{2}}$.

**Figure 4:**
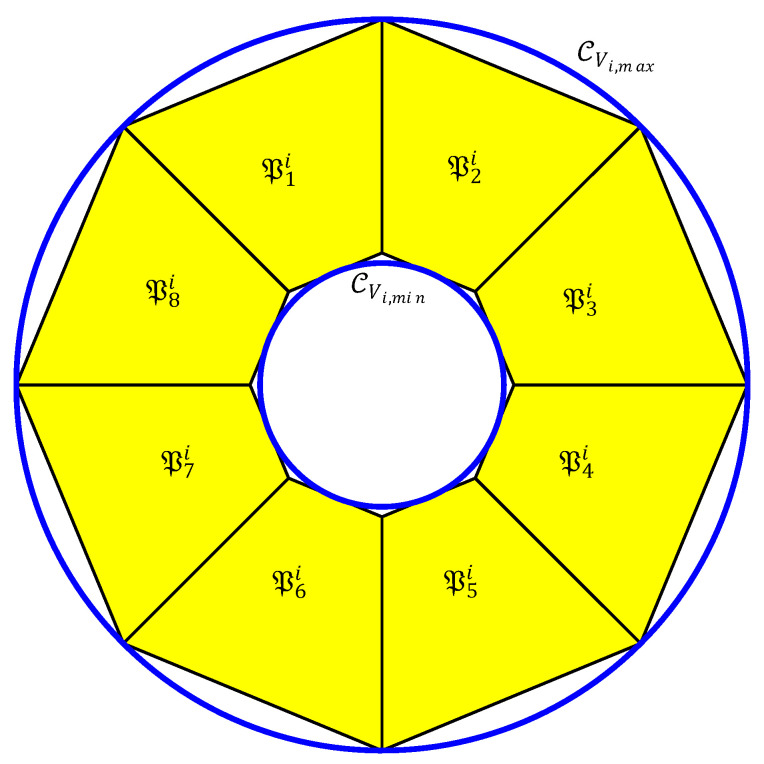
Velocity vector domain and its polytopic approximation. Blue circles represent the maximum and minimum allowed speed.

Equation (16) defines a set of non-convex and nonlinear constraints. In fact, as shown in Figure 4, the velocity vector has always to be kept within the region between two concentric circles $C_{V_{i,min}}$ and $C_{V_{i,max}}$, with radius equal to $V_{i,min}$ and $V_{i,max}$ respectively. For this reason, $C_{V_{i,max}}-C_{V_{i,min}}$ represents the set of admissible $V_{i}$.

In order to define a linear and convex constraint, let consider the two polytopes $P_{V_{i,min}}$ and $P_{V_{i,max}}$ with $n_{v}\geq 3$ vertices, such that $C_{V_{i,min}}$ is inscribed in $P_{V_{min}}^{i}$ and $C_{V_{i,max}}$ is circumscribed at $P_{V_{max}}^{i}$ (see Figure 4). Therefore the set of admissible velocity vector, defined as $C_{V_{i,max}}-C_{V_{i,min}}$, is approximated by the set difference $P_{V_{max}}^{i}-P_{V_{min}}^{i}$.

Let $P^{i}=\left\{P_{s}^{i},\;s=1,2,\ldots ,n_{v}\right\}$ be the set of polytopes needed to define $P_{V_{max}}^{i}-P_{V_{min}}^{i}$ where:

(17) $$P_{s}^{i}=\left\{V_{i}\left(k+p+1|k\right)\in R^{2}:\;\mu_{i,s}^{v}V_{i}\left(k+p+1|k\right)\leq \nu_{i,s}^{v}\right\}$$

with $\mu_{i,s}^{v}$ and $\nu_{i,s}^{v}$ constant matrix and vector, respectively.

Consequently, the set of non-convex linear constraints $V_{i}\left(k+p+1|k\right)\in P^{i}$ can be converted into a convex one, by using the so-called big-M reformulation [83] that results in a MIQP (Mixed Integer Quadratic Programming) reformulation of the problem. This approach requires to increase the number of optimization variables, by introducing $n_{v}$ additional binary variables $\delta_{i,s}^{v}\left(k+p+1|k\right)\in \left\{0,1\right\}$ ($s=1,2\ldots ,n_{v}$), at each time step k+p ($p=0,\ldots ,n_{p}$). Velocity constraints (16) are then reformulated as follows:

(18) $$\left\{\begin{array}{c} \mu_{i,1}^{v}V_{i}\left(k+p+1|k\right)\leq \nu_{i,1}^{v}+M\left(1-\delta_{i,1}^{v}\left(k+p+1|k\right)\right)\\ \mu_{i,2}^{v}V_{i}\left(k+p+1|k\right)\leq \nu_{i,2}^{v}+M\left(1-\delta_{i,2}^{v}\left(k+p+1|k\right)\right)\\ \vdots \\ \mu_{i,n_{v}}^{v}V_{i}\left(k+p+1|k\right)\leq \nu_{i,n_{v}}^{v}+M\left(1-\delta_{i,n_{v}}^{v}\left(k+p+1|k\right)\right)\\ \sum_{s=1}^{n_{v}}\delta_{i,s}^{v}\left(k+p+1|k\right)=1 \end{array}\right.\;\forall p=0,\ldots ,n_{p}$$

where $M={\left[M_{x},M_{y}\right]}^{T}$, $M_{x}$ and $M_{y}$ being sufficiently large numbers.

On the other hand, acceleration constraints can be defined as follows:

(19) $$\left[\begin{array}{c} a_{i,min}^{\|}\left(k\right)\\ a_{i,min}^{⊥}\left(k\right) \end{array}\right]\leq \left[\begin{array}{c} a_{i}^{\|}\left(k+p|k\right)\\ a_{i}^{⊥}\left(k+p|k\right) \end{array}\right]\leq \left[\begin{array}{c} a_{i,max}^{\|}\left(k\right)\\ a_{i,max}^{⊥}\left(k\right) \end{array}\right]\;\forall p=0,\ldots ,n_{p}$$

where superscripts ‖ and ⊥ indicate tangential and normal components to the flight trajectory. The values of maximum and minimum accelerations, $a_{i,min}^{\|}\left(k\right)$, $a_{i,min}^{⊥}\left(k\right)$, $a_{i,max}^{\|}\left(k\right)$, and $a_{i,max}^{⊥}\left(k\right)$, are related to the available thrust and the turn rate capabilities of the aircraft. In particular, $a_{i}^{\|}\left(k\right)$ is produced with the excess of thrust with respect to drag force in the wing levelled forward flight approximation, whereas normal acceleration limits is related to the minimum turning radius $r_{i}$ as follows:

(20) $$a_{i}^{⊥}\left(k\right)\approx \frac{V_{i}{\left(k\right)}^{2}}{r_{i}}$$

In order to obtain linear constraints, accelerations have to be translated into the global reference system (flat Earth-fixed reference frame). Let $T_{i}$ be the rotation matrix from the local reference frame to the global one. Such a matrix is precomputed at time *k* and assumed constant in the prediction horizon $T_{i}\left(k+p|k\right)=T_{i}\left(k|k\right)$. Consequently, the acceleration constraints can be formulated as the following set of linear inequalities:

(21) $$T_{i}\left(k|k\right)\left[\begin{array}{c} a_{i,min}^{\|}\left(k\right)\\ a_{i,min}^{⊥}\left(k\right) \end{array}\right]\leq \left[\begin{array}{c} a_{i,x}\left(k+p|k\right)\\ a_{i,y}\left(k+p|k\right) \end{array}\right]\leq T_{i}\left(k|k\right)\left[\begin{array}{c} a_{i,max}^{\|}\left(k\right)\\ a_{i,max}^{⊥}\left(k\right) \end{array}\right]\;\forall p=0,\ldots ,n_{p}$$

Let’s finally define an enlarged vector of design variables $\Omega_{i}\left(k\right)={\left[u_{i}{\left(k|k\right)}^{T},\ldots ,u_{i}{\left(k+n_{c}|k\right)}^{T},\delta_{i,s}^{v}\left(k+1|k\right),\ldots ,\delta_{i,s}^{v}\left(k+n_{p}+1|k\right)\right]}^{T}$. Problem 1 is then reformulated as follows:

**Problem** **2.** *At each time step k, find the optimal sequence of design variables $\Omega_{i}\left(k\right)$ such that it minimizes the cost function (15), in presence of linear constraints (18) and (21).*

Problem 2 turns out to be a Mixed Integer Quadratic Programming problem, which has efficient numerical solvers available [84,85].

According to the receding horizon paradigm, the output of the MPC is the control signal at p=0, $u_{i}^{*}\left(k|k\right)={\left[a_{i,x}^{*}\left(k|k\right),a_{i,y}^{*}\left(k|k\right)\right]}^{T}$.

The OPP block computes the speed change $u_{V_{i}}$ and the turn rate $u_{\psi_{i}}$ to be passed to the aircraft FCS as follows:

(22) $$\begin{array}{c} u_{V_{i}}\left(k\right)=\frac{2v_{i,x}\left(k\right)a_{i,x}^{*}\left(k|k\right)+2v_{i,y}\left(k\right)a_{i,y}^{*}\left(k|k\right)}{\sqrt{v_{i,x}{\left(k\right)}^{2}+v_{i,y}{\left(k\right)}^{2}}}\\ u_{\psi_{i}}\left(k\right)=\frac{v_{i,x}\left(k\right)a_{i,y}^{*}\left(k|k\right)-v_{i,y}\left(k\right)a_{i,x}^{*}\left(k|k\right)}{v_{i,x}{\left(k\right)}^{2}+v_{i,y}{\left(k\right)}^{2}} \end{array}$$

### 5.2. Collision Avoidance Algorithm and Constraints

The Collision Avoidance Algorithm (CAA) is designed to guarantee that each aircraft in the formation is able to avoid collision with the other vehicles or with any existing obstacle.

Consider $N_{o}=N_{ob}+\left(N-1\right)$ equivalent obstacles $C_{j}\left(k\right)$ ($N_{ob}$ real obstacles and N−1 aircraft of the swarm). Assume that each obstacle *j* is modeled as a circumference of radius ${\bar{r}}_{j}$, centered at point ${\bar{C}}_{j}\left(k\right)={\left[{\bar{x}}_{j}\left(k\right),{\bar{y}}_{j}\left(k\right)\right]}^{T}$.

The CAA verifies if any distance between the *i*-th aircraft and the obstacles is less than or equal to a fixed safety distance $d_{safety}$, evaluating the set of possible colliding obstacles $O_{i}\left(k\right)$.

**Definition** **5.** *The set of possible colliding obstacles $O_{i}\left(k\right)$ at time k is defined as:*

(23) $$O_{i}\left(k\right)=\left\{\forall C_{j}\left(k\right):\left|P_{i}\left(k\right)-{\bar{C}}_{j}\left(k\right)\right|-{\bar{r}}_{j}-{\bar{r}}_{i}\leq d_{safety}\right\}$$

*where ${\bar{r}}_{i}$ and ${\bar{r}}_{j}$ are the equivalent radius of the i-th aircraft and j-th obstacle respectively.*

In accordance with the reactive scheme [86,87], only the nearest colliding obstacle within set $O_{i}\left(k\right)$ is taken into account by the MPC [8,78] by adding a limited number of constraints to the Problem 2.

**Figure 5:**
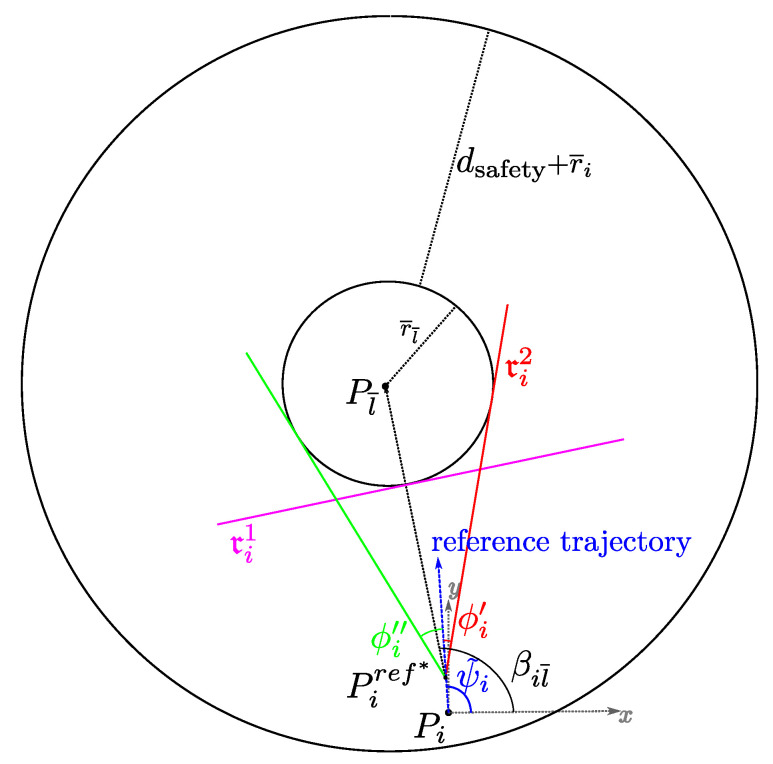
Collision Avoidance constraints (Time information has been omitted). Obstacle is described by circumference centered in the point $P_{j}$; tangent lines $r_{i}^{1}$ and $r_{i}^{2}$ define the admissible region for the *i*-th future position.

Without loss of generality, assume that the forthcoming potential collision, for the vehicle *i*, is going to occur with the aircraft $\bar{l}$, as shown in Figure 5.

For all the time instants in the prediction horizon k+p ($p=1,\ldots ,n_{p}$), the UAV position is constrained to fall within a region delimited by two straight lines. This guarantees the desired safety distance $d_{safety}$.

The first boundary line $r_{i}^{1}\left(k+p|k\right)$ is normal to the vector $P_{\bar{l}}\left(k+p|k\right)-P_{i}\left(k+p|k\right)$ and tangent to the circumference $C_{\bar{l}}$ with radius ${\bar{r}}_{\bar{l}}$, centered in the predicted position $P_{\bar{l}}\left(k+p|k\right)={\left[{\hat{x}}_{\bar{l}}\left(k+p|k\right),{\hat{y}}_{\bar{l}}\left(k+p|k\right)\right]}^{T}$.

Since there is no cooperation between vehicles, the obstacle future positions $P_{\bar{l}}\left(k+p|k\right)={\left[{\hat{x}}_{\bar{l}}\left(k+p|k\right),{\hat{y}}_{\bar{l}}\left(k+p|k\right)\right]}^{T}$, over the prediction horizon ($p=1,\ldots ,n_{p}+1$), are estimated assuming that both speed and heading are constant, i.e. $V_{\bar{l}}\left(k+p|k\right)=V_{\bar{l}}\left(k|k\right)$, $\psi_{\bar{l}}\left(k+p|k\right)=\psi_{\bar{l}}\left(k|k\right)$.

Line $r_{i}^{1}\left(k+p|k\right)$ is defined as follows:

(24) $$r_{i}^{1}\left(k+p|k\right):c_{\alpha ,i}\left(k+p|k\right)\left(x-{\hat{x}}_{\bar{l}}\left(k+p|k\right)\right)+s_{\alpha ,i}\left(k+p|k\right)\left(y-{\hat{y}}_{\bar{l}}\left(k+p|k\right)\right)-{\bar{r}}_{\bar{l}}=0$$

with

(25) $${\left[c_{\alpha ,i}\left(k+p|k\right),s_{\alpha ,i}\left(k+p|k\right)\right]}^{T}=\frac{P_{\bar{l}}\left(k+p|k\right)-P_{i}\left(k\right)}{\left|\right|P_{\bar{l}}\left(k+p|k\right)-P_{i}\left(k\right)\left|\right|}$$

Another line delimiting the region that can be occupied by the aircraft trajectory is that reported as $r_{i}^{2}\left(k+p|k\right)$ in Figure 5. This is tangent to the obstacle $C_{\bar{l}}$ and passes through a point $P_{i}^{ref^{*}}=P_{i}^{ref}\left(k+p^{*}|k\right)=\left(x_{i}^{ref}\left(k+p^{*}|k\right),y_{i}^{ref}\left(k+p^{*}|k\right)\right)$, on the reference trajectory ($p^{*}\geq 1$), which is ahead the current aircraft position. This choice to look forward gives enough space and time to maneuver in order to avoid the forbidden regions.

For each time step k+p ($p=1,\ldots ,n_{p}$), consider the vector $P_{\bar{l}}\left(k+p|k\right)-P_{i}^{ref}\left(k+p^{*}|k\right)$. Its direction with respect to the *x* axis is defined by the angle $\beta_{i\bar{l}}\left(k+p|k\right)$.

Compute the angles:

$$\phi_{i}^{\prime }\left(k+p|k\right)=-\arcsin({\bar{r}}_{\bar{l}}/\left|\right|P_{\bar{l}}\left(k+p|k\right)-P_{i}^{ref}\left(k+p^{*}|k\right)\left||\right)$$

$$\phi_{i}{}^{\prime \prime }\left(k+p|k\right)=\arcsin({\bar{r}}_{\bar{l}}/\left|\right|P_{\bar{l}}\left(k+p|k\right)-P_{i}^{ref}\left(k+p^{*}|k\right)||)\;.$$

The CAA algorithm selects the angle $\phi_{i}\left(k+p|k\right)\in \left\{\phi_{i}^{\prime }\left(k+p|k\right),\phi_{i}"\left(k+p|k\right)\right\}$ minimizing the deviation from the desired heading ${\tilde{\psi }}_{i}\left(k\right)$.

Line $r_{i}^{2}\left(k+p|k\right)$ is defined as follows

(26) $$r_{i}^{2}\left(k+p|k\right):s_{\theta ,i}\left(k+p|k\right)\left(x-x_{i}^{ref}\left(k+p^{*}|k\right)\right)-c_{\theta ,i}\left(k+p|k\right)\left(y-y_{i}^{ref}\left(k+p^{*}|k\right)\right)=0$$

where $c_{\theta ,i}\left(k+p|k\right)=\cos\left(\theta_{i}\left(k+p|k\right)\right)$, and $s_{\theta ,i}\left(k+p|k\right)=\sin\left(\theta_{i}\left(k+p|k\right)\right)$, with $\theta_{i}\left(k+p|k\right)={\left\lfloor \beta_{i\bar{l}}\left(k+p|k\right)+\phi_{i}\left(k+p|k\right)\right\rfloor }_{-\pi }^{\pi }$

It is worth noticing that constraints defined by $r_{i}^{2}\left(k+p|k\right)$ are not strictly needed to perform collision avoidance which could be managed in terms of speed optimization. However this kind of constraint, increases the overall performance of the algorithm implementing the anti-collision action well in advance with respect to the approaching obstacle. For example, without this constraint, in a symmetric condition, with the aircraft reference trajectory pointing to the center of the obstacle, the vehicle would tend to decrease its speed to the minimum, before performing a turn induced by the potential action spreading from the obstacle.

On the other hand, near the obstacle, this constraint may lead to an unfeasible problem being the aircraft initial position not in the feasible region. For this reason, the boundary line $r_{i}^{2}\left(k+p|k\right)$ is deactivated under certain conditions according to the following activation operator:

(27) $$\Lambda_{i,\bar{l}}\left(k+p|k\right)=\left\{\begin{array}{cc} 1\; & \mathrm{if}\;|\beta_{i\bar{l}}\left(k+p|k\right)-\psi_{i}\left(k\right)|\leq \pi /3\\ 0\; & \mathrm{otherwise} \end{array}\right.$$

The following equations define the admissible region over the prediction horizon $\left[k,k+n_{p}\right]$ as constraints to be added to the MPC Problem:

(28) $$\begin{array}{c} c_{\alpha ,i}\left(k+p|k\right)\left(x-{\hat{x}}_{\bar{l}}\left(k+p|k\right)\right)+s_{\alpha ,i}\left(k+p|k\right)\left(y-{\hat{y}}_{\bar{l}}\left(k+p|k\right)\right)-{\bar{r}}_{\bar{l}}=0 \end{array}$$

(29) $$\begin{array}{c} \Lambda_{i,\bar{l}}\left(k+p|k\right)\left(s_{\theta ,i}\left(k+p|k\right)\left(x-{\hat{x}}_{i}^{ref}\left(k+p^{*}|k\right)\right)-c_{\theta ,i}\left(k+p|k\right)\left(y-{\hat{y}}_{i}^{ref}\left(k+p^{*}|k\right)\right)\right)\leq 0 \end{array}$$

for each time step k+p, with $p=0,\ldots ,n_{p}$.

## 6. Numerical Results

To test and validate the proposed guidance system architecture, several numerical simulations were carried out using an ad-hoc simulator developed in a Matlab/Simulink environment. All of the operational scenarios are compliant with homogeneous swarms of micro/mini quadrotor-type UAVs having 10–15 min of flight endurance [88]. The proposed procedure was implemented using Operator Splitting Quadratic Program (OSQP) [89,90] to solve the MPC optimization problem.

The simulation parameters are presented in Table 1.

The position of each aircraft is monitored, checking if collisions are avoided and if the shape of the flight formation is re-established after passing obstacles. A numerical indicator, namely $d_{\mathrm{mean}}$, is also calculated to check the algorithm effectiveness in keeping the swarm on a triangular mesh. It represents the average value of the distance between every pair of visible aircraft in the Delaunay triangulation and is defined as:

(30) $$d_{\mathrm{mean}}\left(k\right)=\frac{\sum_{i=1}^{N}\sum_{j\in {\bar{Q}}_{i}\left(k\right)}d_{i,j}\left(k\right)}{\sum_{i=1}^{N}\mathrm{card}\left({\bar{Q}}_{i}\left(k\right)\right)}$$

To highlight the features of the proposed guidance algorithm, three scenarios were considered. Each scenario lies in a box of 1×1 km, simulating an environment with different obstacle arrangement and size.

Results with three, five and 10 flying vehicles are presented in Scenario #1, and 10 vehicles are used in Scenarios #2 and #3.

Aircraft initial conditions were assigned such that the swarm initial mesh is not composed of equilateral triangles as desired. Simulations were repeated 10 times for each scenario to avoid different initial conditions affecting the analysis of the results.

### 6.1. Scenario #1—Three Aircraft

**Figure 6:**
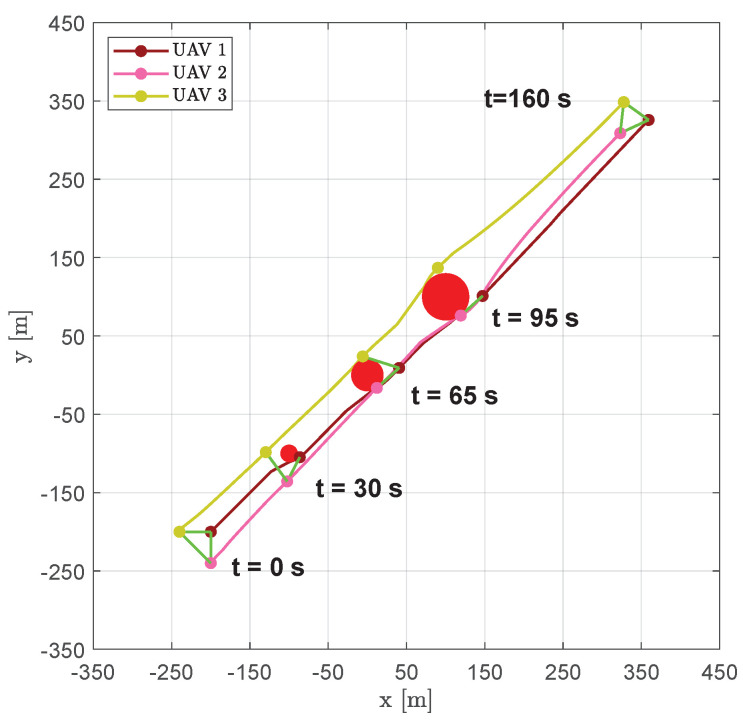
Scenario #1—three aircraft: UAVs’ trajectories. Red circles represent the obstacles; colored markers indicate positions at different time instants; green straight lines highlight DTG arcs.

**Figure 7:**
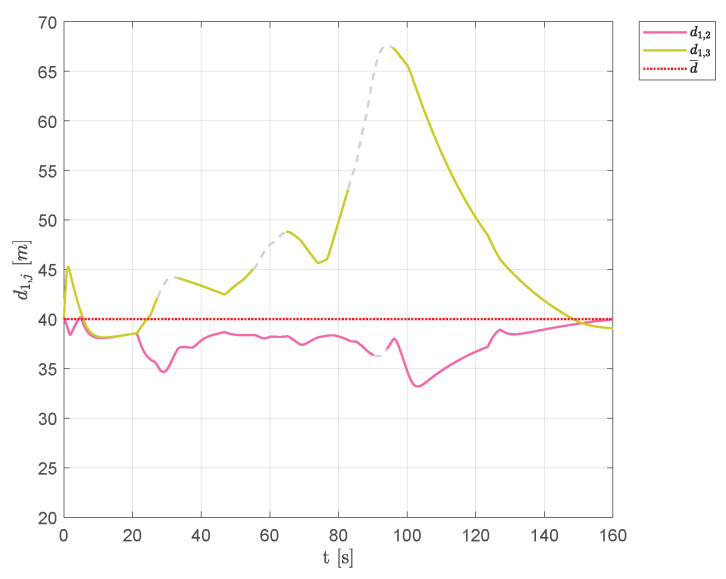
Scenario #1—three aircraft: distances between UAV 1 and the other swarm vehicles. Solid lines are used for visible aircraft, dashed gray lines are employed whenever they are no longer visible. The desired distance is highlighted using a dotted red line.

The first scenario is made up of three circular obstacles of different sizes, placed between the fleet’s starting position and the target point $P_{f}$. Figure 6 shows the resulting trajectories obtained involving three aircraft; markers are used to show vehicles positions at the fixed time instants t=0 s, t=30 s, t=65 s, t=95 s and t=160 s, whereas green lines are used to highlight the DTG arcs. Figure 7 shows the distances between vehicle 1 and the other aircraft of the swarm. Solid lines indicate the distances from vehicle 1 from any other visible aircraft; whenever the aircraft are no longer visible dashed gray lines are used.

**Figure 8:**
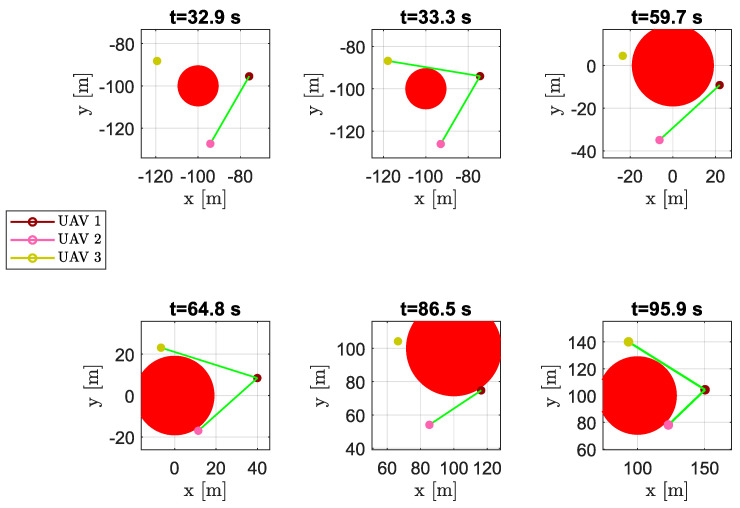
Scenario #1—three aircraft: snapshots of UAVs’ positions at different time instants. Colored markers indicate UAVs’ actual positions; straight green lines show the DTG arcs. Several swarm configurations during obstacle avoidance are highlighted.

During the flight, in presence of obstacles, the swarm split into two parts (see aircraft positions at time instants t=32.9s, t=59.7s, and t=86.5s in Figure 8). Since aircraft 1 and 2 overcame the obstacles, passing on the same side, their mutual distance $d_{1,2}$ was always close to the desired distance. On the other hand, aircraft 3 passed on the other side of the obstacles and, consequently, distances $d_{1,3}$ and $d_{2,3}$ greatly deviated from the desired distance $\bar{d}$ near the obstacles. The goal of keeping a uniform distance between aircraft was achieved while avoiding any collision. As we can see, vehicle 2 almost always belonged to ${\bar{Q}}_{1}$, the set of visible aircraft from vehicle 1. Vehicle 3, passing on the other side of the obstacles, became not visible from aircraft 1 during some time intervals. However, after passing the obstacles (t≥95.9s), the DTG was newly connected and aircraft tended to restore an equilateral triangles-based flight formation with edges equal to the desired distance $\bar{d}$.

**Figure 9:**
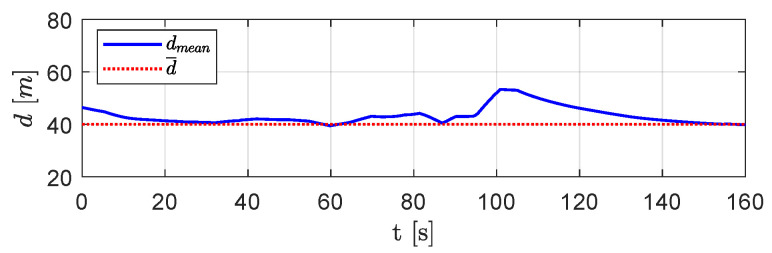
Scenario #1—three aircraft: mean distance between visible aircraft (blue solid line) compared with desired distance (red dotted line).

Figure 9 shows the mean distance $d_{mean}$. As expected, this distance increased with respect to the desired one only in short time intervals, where the presence of obstacles led the aircraft to deviate more from the desired mutual distances.

**Figure 10:**
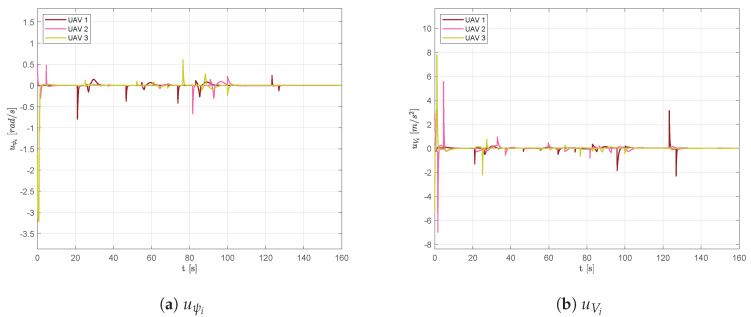
Scenario #1—three aircraft: control signal computed by TTCAA.

Time histories of the control signals $u_{\psi }$ and $u_{V}$ are presented in Figure 10. It is worth noticing that at the beginning of the simulation, the random initial position of the aircraft required them to accelerate and turn to establish the desired swarm shape. Furthermore, control signals presented peaks before passing obstacles at the activation of the collision avoidance and, after that, in order to re-compose the swarm.

### 6.2. Scenario #1—Five Aircraft

**Figure 11:**
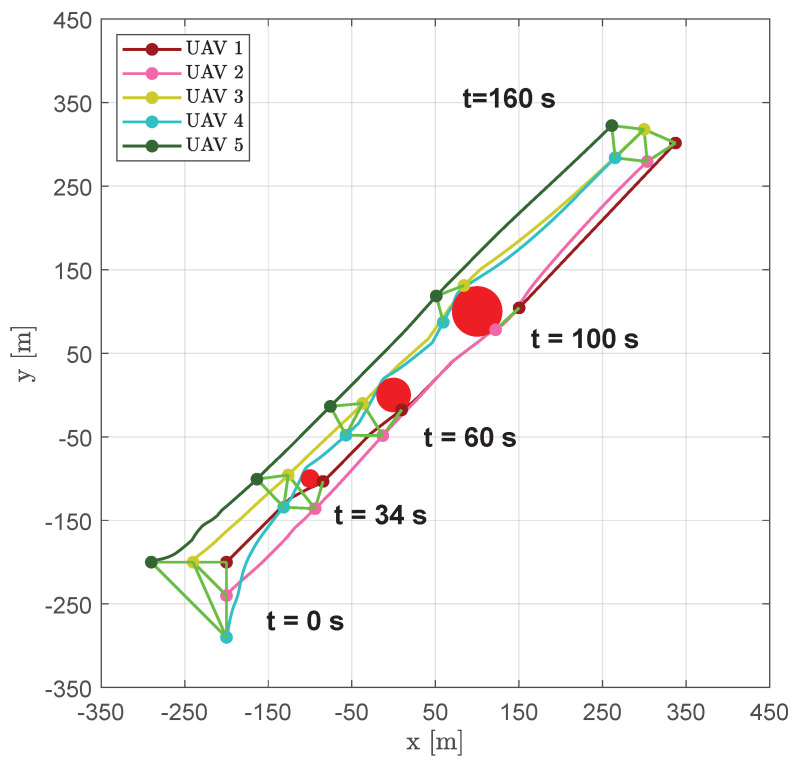
Scenario #1—five aircraft: UAVs trajectories. Red circles represent obstacles; colored markers indicate positions at different time instants; green straight lines highlight DTG arcs.

The goal of keeping a uniform distance between aircraft was also achieved with a higher number of vehicles. Figure 11 shows the resulting trajectories obtained with five aircraft together with their positions (markers) and the DTG edges (green lines) at several time instants (t=0 s, t=34 s, t=60 s, t=100 s and t=160 s).

**Figure 12:**
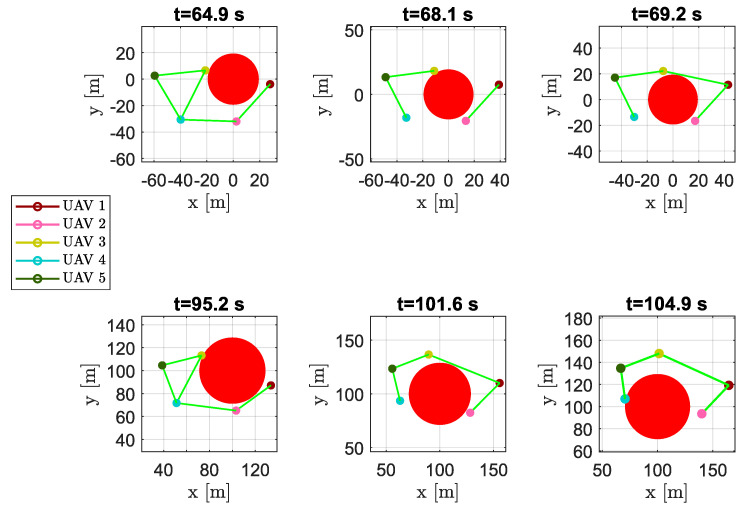
Scenario #1—five aircraft: snapshots of UAVs positions at different time instants. Colored markers indicate UAVs actual positions; straight green lines show the DTG arcs. During obstacle avoidance the DTG may became not connected, separating the formation into two distinct sub-graphs.

As shown in Figure 12, near obstacles the formation split into two parts, passing at the opposite sides of the obstacles; e.g., at time instant t=68.1s the DTG was not connected and was composed of two distinct sub-graphs made up of vehicles {3,4,5} and {1,2}, seperately.

**Figure 13:**
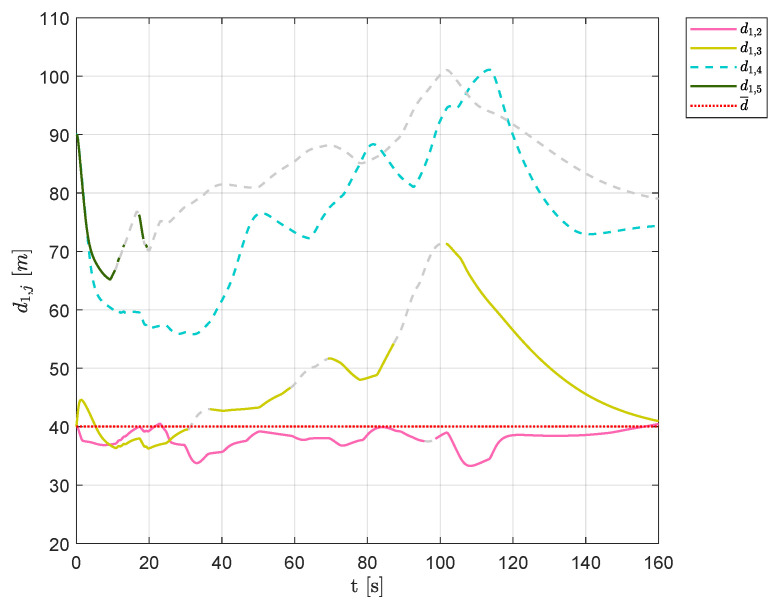
Scenario #1—five aircraft: distances between UAV 1 and the other swarm vehicles. Solid lines are used for visible aircraft, dashed gray lines are employed whenever they are no longer visible, whereas dashed colored lines are used for never visible UAVs. The desired distance is highlighted using a dotted red line.

In Figure 13 the distances between vehicle 1 and the others are shown. Aircraft 4 never belonged to the set of vehicles visible from UAV 1, ${\bar{Q}}_{1}$, resulting in a distance $d_{1,4}$ (indicated with a dashed line) that was always greater than the desired distance. On the other hand, aircraft 2 was almost always visible from UAV 1, resulting in a distance approximately equal to the desired one during the flight, except in short time intervals, where collision avoidance prevented vehicles from the desired relative positioning. Vehicle 3, while passing on the opposite side of obstacles with respect to aircraft 1, tried to re-compose the swarm after them. Finally, at t>100s, $d_{1,3}$ clearly tended to the desired distance $\bar{d}$.

It is worth noting that DTG, and consequently the swarm configuration, changes over time: for instance, at t≤10 s, as shown in Figure 13, aircraft 1 belonged to two triangles, one with vehicles 3 and 5 and the other with 2 and 3, while at approximately t>100 s it belonged only to a triangle with 2 and 3.

**Figure 14:**
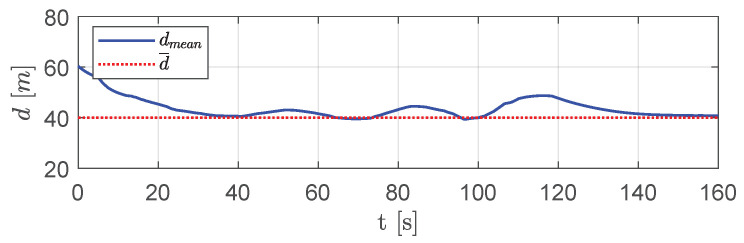
Scenario #1—five aircraft: mean distance between visible aircraft (blue solid line) compared with desired distance (dotted red line).

Figure 14 shows the mean distance $d_{mean}$ between vehicles over DTG. Although such a distance showed slight deviations in short time intervals, due to a change in swarm configuration in the presence of obstacles, it tended always to the desired distance $\bar{d}$.

### 6.3. Scenario #1—10 Aircraft

**Figure 15:**
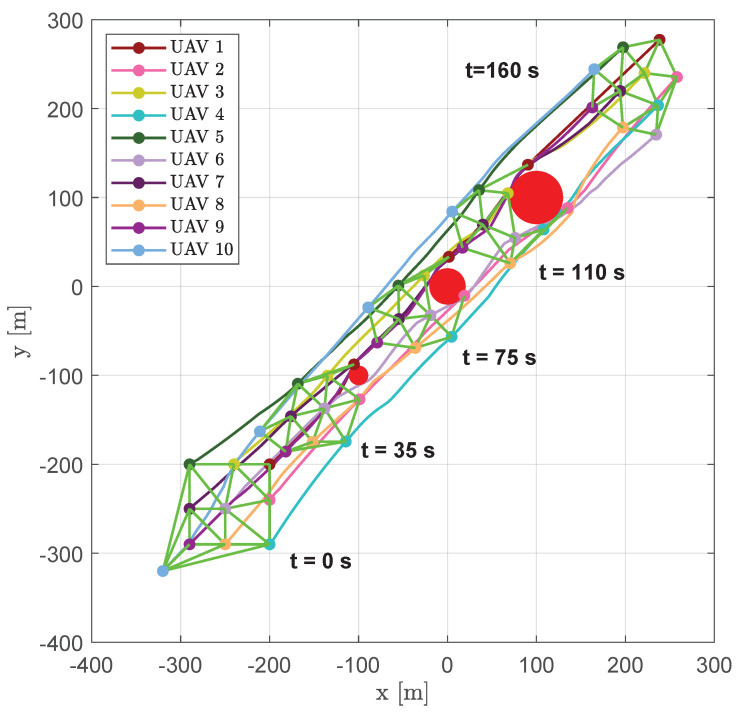
Scenario #1—10 aircraft: UAV trajectories. Red circles represent obstacles; colored markers indicate positions at different time instants; green straight lines highlight DTG arcs.

Figure 15 shows the trajectories of a swarm of 10 aircraft, highlighting their positions (markers) and edges of DTG (green lines) at several time instants (t=0 s, t=35 s, t=75 s, t=110 s and t=160 s).

**Figure 16:**
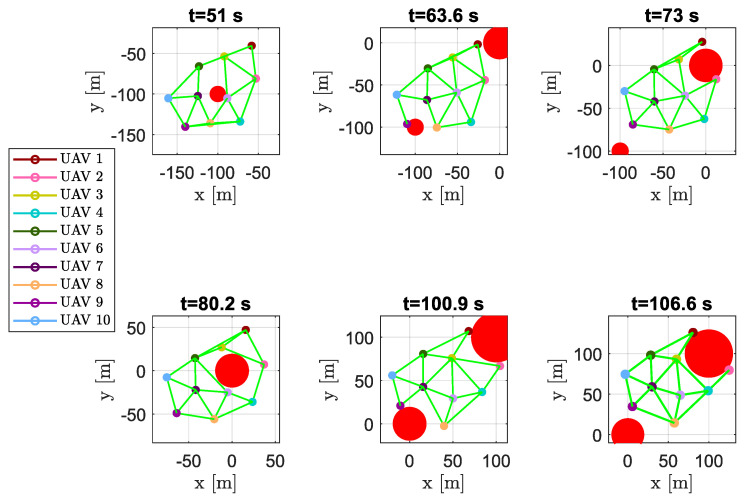
Scenario #1—10 aircraft: snapshots of aircraft positions at different time instants. Colored markers indicate UAVs’ current positions; straight green lines show the arcs of modified DTG. Chosen time instants highlight swarm connectivity during obstacle avoidance.

In Figure 16, snapshots of swarm configuration at time instants t=51 s, t=63.6 s, t=73 s, t=80.2 s, t=100.9 s and t=106.6 s are shown. It is worth noting that, although the vehicles passed on both sides of the obstacles, virtually splitting the swarm in two parts, the DTG remained connected, giving the ability to create a regular mesh also around the obstacles.

**Figure 17:**
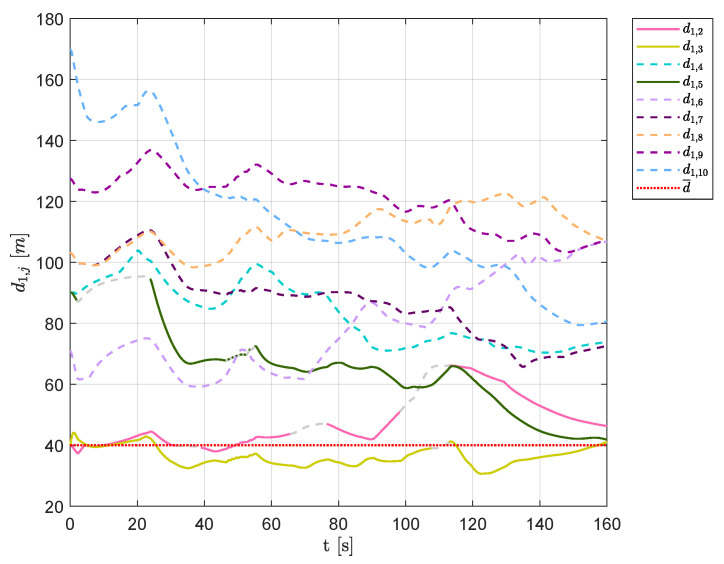
Scenario #1—10 aircraft: distances between UAV 1 and the other swarm vehicles. Solid lines are used for visible aircraft and dashed gray lines are employed whenever they are no longer visible, whereas dashed colored lines are used for never visible UAVs. The desired distance is highlighted using a dotted red line.

In Figure 17, the distances of each vehicle from aircraft 1 are shown, highlighting that UAVs 4, 6, 7, 8, 9 and 10 never belonged to ${\bar{Q}}_{1}$ (their distances are represented as colored dashed lines), whereas the remaining aircraft showed a continuously changing swarm configuration, finding an equilibrium only after the last obstacle (t>120 s).

**Figure 18:**
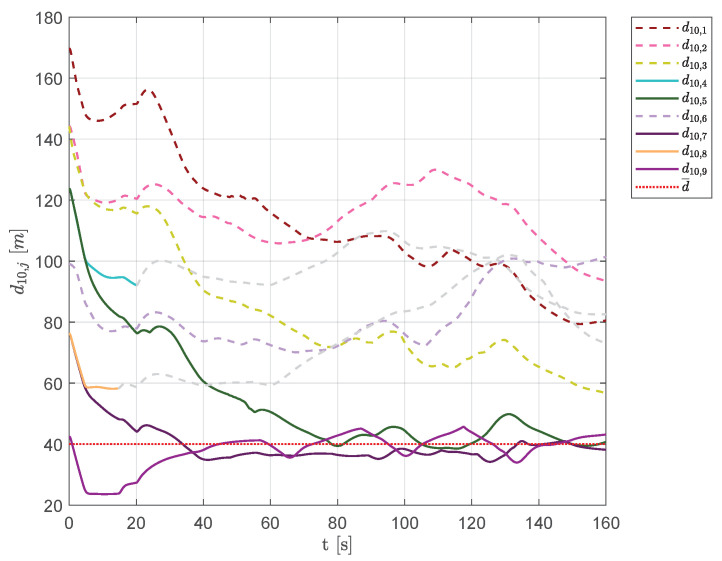
Scenario #1—10 aircraft: distances between UAV 10 and the other swarm vehicles. Solid lines are used for visible aircraft and dashed gray lines are employed whenever they are no longer visible, whereas dashed colored lines are used for never visible UAVs. The desired distance is highlighted using a dotted red line.

Figure 18 shows the distances of each vehicle from aircraft 10. Vehicles 5, 7 and 9 always belonged to ${\bar{Q}}_{10}$ (their distances are represented as solid lines), whereas aircraft 4 and 8 belonged to the set of visible neighbors only at the beginning of the simulation.

Looking at Figure 17 and Figure 18, it is clear that aircraft tried to compose regular triangle-based sub-swarms with visible neighbors, also during the obstacle avoidance, maintaining the mutual distances as equally as possible to the desired distance.

**Figure 19:**
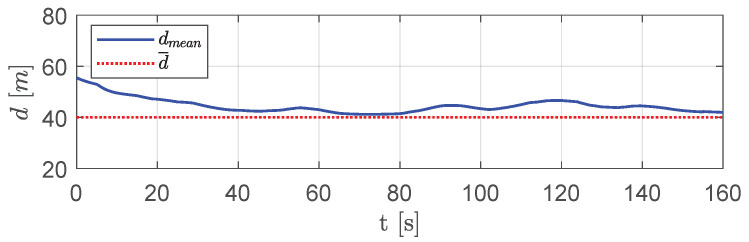
Scenario #1—10 aircraft: mean distance between visible aircraft (blue solid line), compared with desired distance (red dotted line).

Figure 19 presents the average distance between visible aircraft. As shown, aircraft were able to keep a mutual average distance very close to the desired one with slight deviations only when overcoming obstacles.

### 6.4. Scenario #2—10 Aircraft

Scenario #2 is made up of four larger obstacles placed between the starting and the target points, in order to spread the swarm before a narrow channel that forces the aircraft to re-group. After that, the remaining big obstacle forces the swarm to divide again before reaching the target. This kind of scenario is an interesting test to stress the collision avoidance algorithm and prove the swarm’s capability to rendezvous after the obstacles. Any sub-swarm, due to the presence of the obstacles, is automatically re-linked to the overall DTG to re-estabilish the aircraft formation.

**Figure 20:**
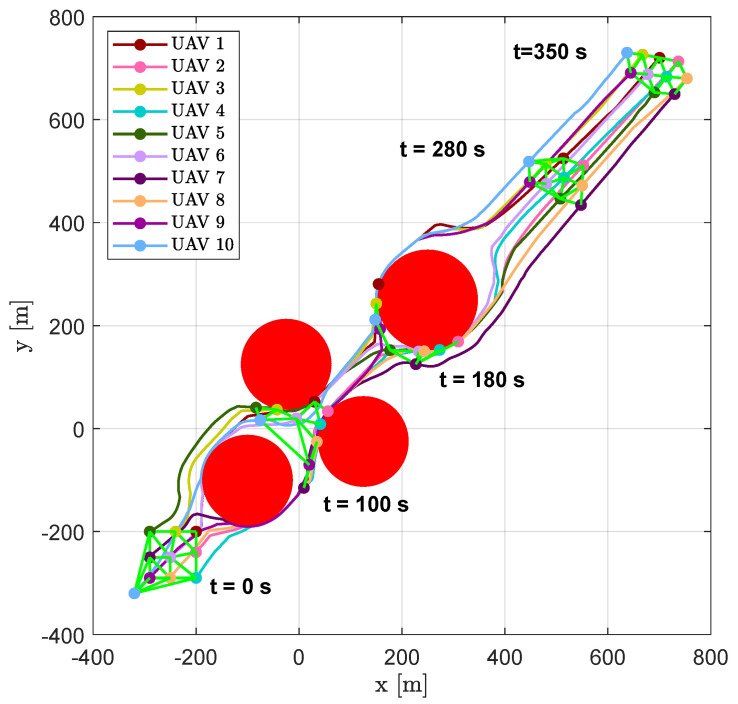
Scenario #2—10 aircraft: UAV trajectories. Red circles represent obstacles; colored markers indicate positions at different time instants; green straight lines highlight DTG arcs.

Figure 20 shows trajectories of the vehicles, highlighting the positions and the DTG at several time instants (t=0 s, t=100 s, t=180 s, t=280 s and t=350 s).

**Figure 21:**
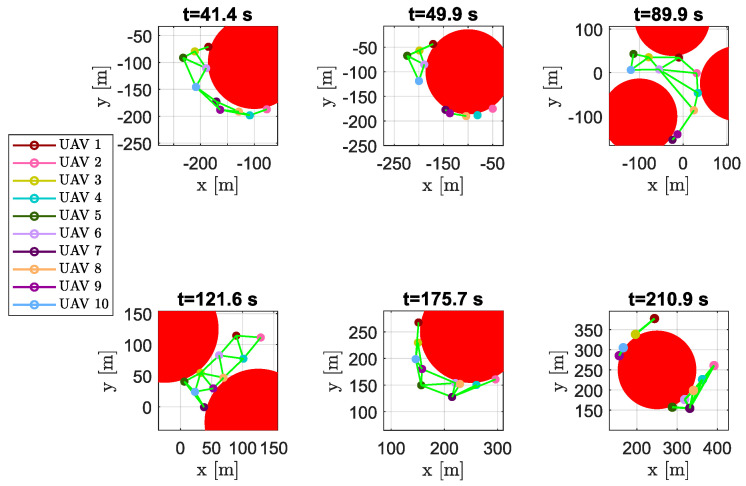
Scenario #2—10 aircraft: snapshots of UAVs’ positions at different time instants. Colored markers indicate UAVs’ actual positions; straight green lines show the DTG arcs. Chosen time instants highlight how the swarm is able to adapt its shape to the environment. At time instants t=49.9 s and t=210.9 s the DTG was not connected, and the swarm split into several sub-swarms.

As expected, after the first obstacle, the swarm divided into four sub-swarms, composed of vehicles {1,3,5,6,10}, {7,8,9}, {2} and {4} (see aircraft positions at time instant t=49.9 s in Figure 21). To pass the channel between the following obstacles the swarm had to re-organize its shape (see the snapshots at time instants t=89.9 s and t=121.6 s in Figure 21). After that, another split happened in order to overcome the last obstacle; the new sub-swarms, composed of vehicles {2,4,5,6,7,8} and {1,3,9,10}, merged at the end of the obstacle avoidance. The result highlights the freedom of the swarm to compose different sub-swarms without assigning a prescribed structure, which is difficult to obtain using a leader (or virtual leader)–follower paradigm.

**Figure 22:**
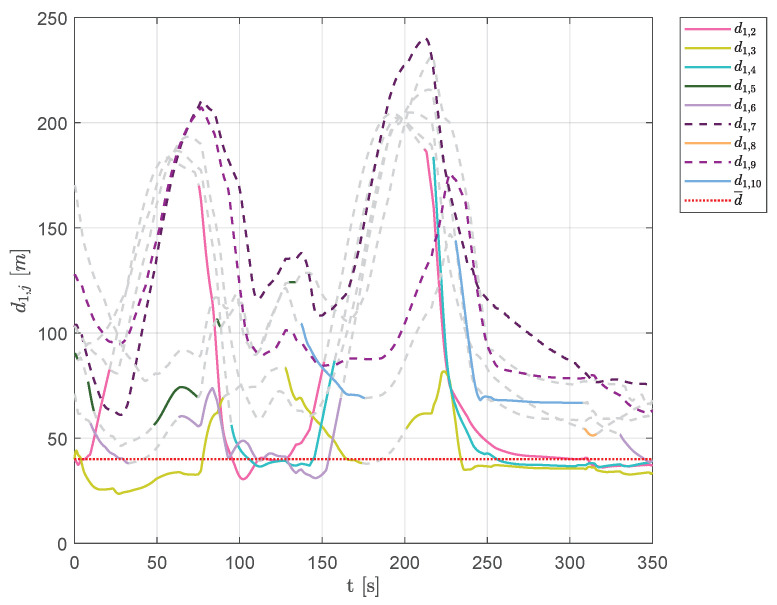
Scenario #2—10 aircraft: distances between UAV 1 and the other swarm vehicles. Solid lines are used for visible aircraft and dashed gray lines are employed whenever they are no longer visible, whereas dashed colored lines are used for never-visible UAVs. The desired distance is highlighted using a dotted red line.

**Figure 23:**
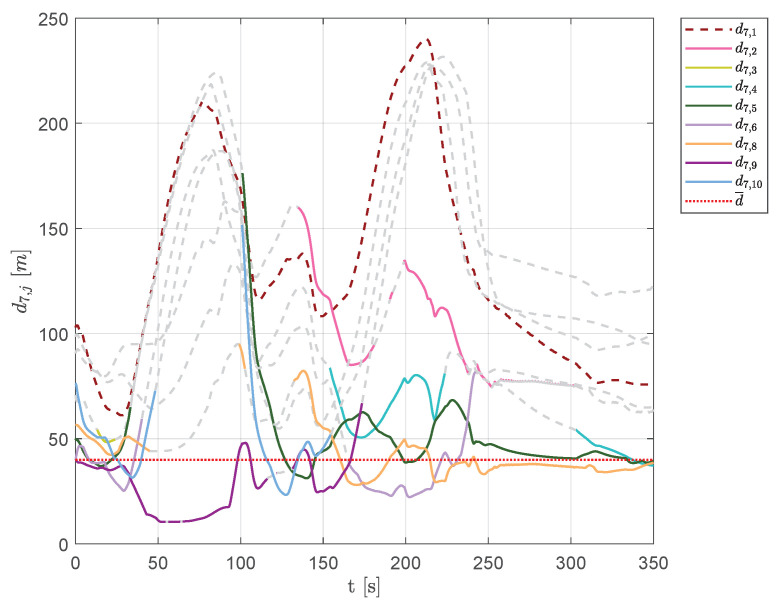
Scenario #2—10 aircraft: distances between UAV 7 and the other swarm vehicles. Solid lines are used for visible aircraftand dashed gray lines are employed whenever they are no longer visible, whereas dashed colored lines are used for never-visible UAVs. The desired distance is highlighted using a dotted red line.

Figure 22 and Figure 23 show the aircraft distances from aircraft 1 and 7. A continuous change of swarm configuration can be noticed from the repeated switching between solid and dashed lines.

**Figure 24:**
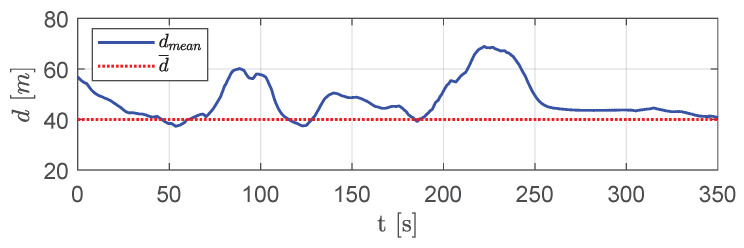
Scenario #2—10 aircraft: mean distance between visible aircraft (blue solid line) compared with desired distance (red dotted line).

It is worth noting that, after obstacles, the swarm tended to a configuration with mutual distances between visible aircraft that was equal to the desired one, e.g., aircraft 1 created equilateral triangles with aircraft 2, 3 and 4. This trend is confirmed by Figure 24, which shows the average distance between neighboring visible aircraft.

### 6.5. Scenario #3—10 Aircraft

The third scenario allows to prove the capability of the swarm to rapidly adapt its shape to the environment. The first group of obstacles consists of two large circles, creating a narrow corridor before the third obstacle, which is placed to force the swarm to spread. The second group is composed of a full area of smaller obstacles forcing the swarm to split into several sub-swarms.

**Figure 25:**
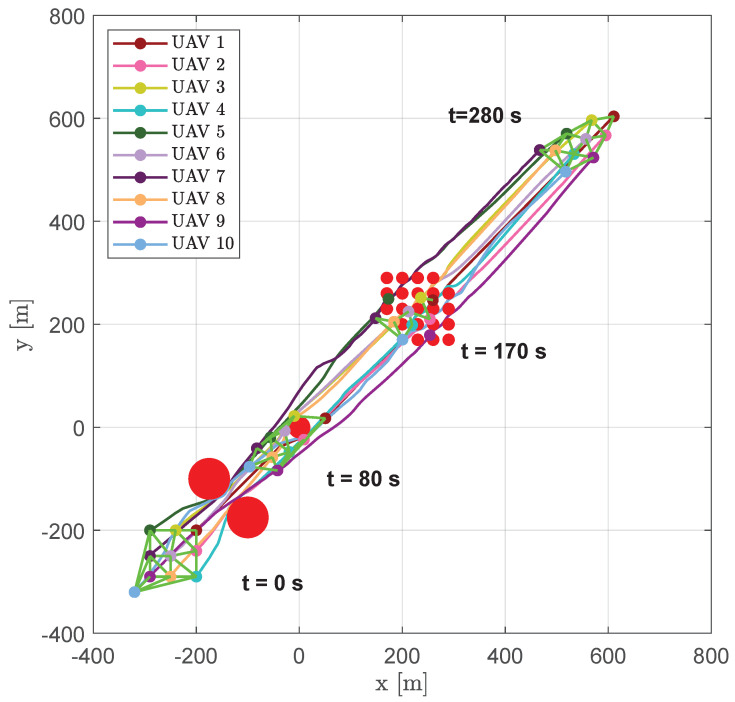
Scenario #3—10 aircraft: UAV trajectories. Red circles represent obstacles; colored markers indicate the UAVs positions at different time instants; green straight lines highlight DTG arcs.

Figure 25 shows trajectories of the vehicles, highlighting their positions and the DTG arcs at several time instants (t=0 s, t=80 s, t=170 s, and t=280 s).

**Figure 26:**
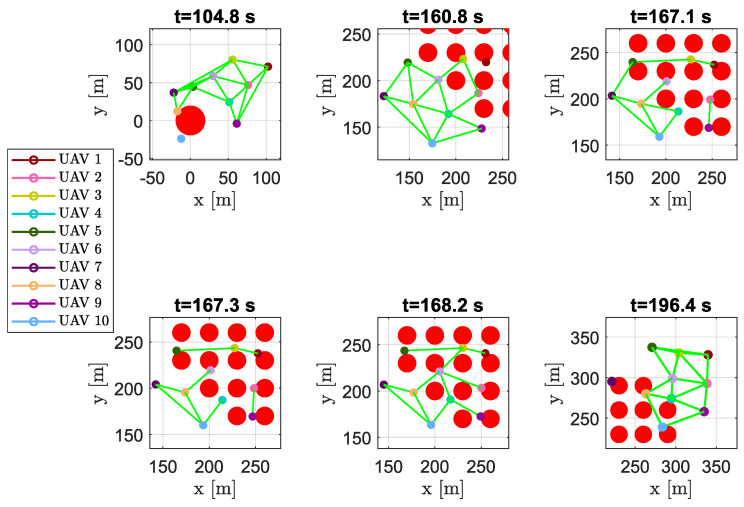
Scenario #3—10 aircraft: snapshots of UAV positions at different time instants. Colored markers indicate UAVs’ actual positions, straight green lines show the DTG arcs. Snapshots highlight how the DTG connectivity rapidly changes in presence of obstacles.

At the beginning of the simulation, the swarm was forced to compact itself to pass between two big obstacles simulating the presence of a narrow crossing channel; then, the reduced size of the third obstacle permited the swarm to recompose a compact shape and pass it, losing the DTG connectivity at some time instants, as reported in Figure 26 at t=104.8 s. Once aircraft reached the area full of obstacles, they scattered in order to overcome the obstacles, creating several time-varying sub-swarms as highlighted in Figure 26 at t∈[160.8,196.4] s.

**Figure 27:**
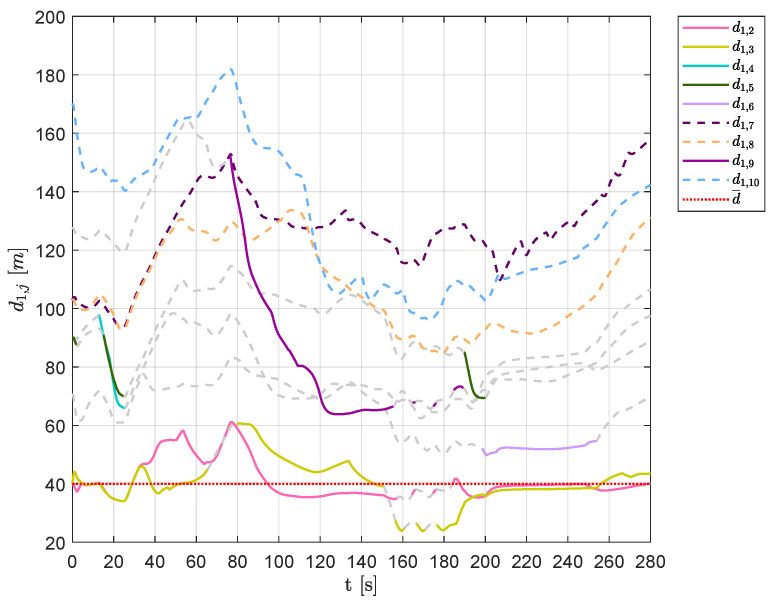
Scenario #3—10 aircraft: distances between UAV 1 and the other swarm vehicles. Solid lines are used for visible aircraft and dashed gray lines are employed whenever they are no longer visible, whereas dashed colored lines are used for never visible UAVs. The desired distance is highlighted using a dotted red line.

**Figure 28:**
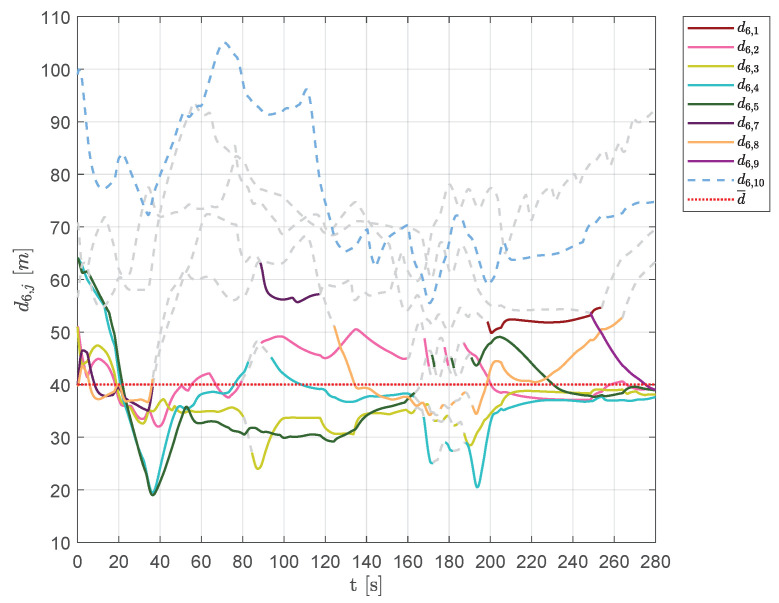
Scenario #3—10 aircraft: distances between UAV 6 and the other swarm vehicles. Solid lines are used for visible aircraft and dashed gray lines are employed whenever they are no longer visible, whereas dashed colored lines are used for never visible UAVs. The desired distance is highlighted using a dotted red line.

This behavior is also confirmed by Figure 27 and Figure 28, which show the distances of each vehicle visible from aircraft 1 and 6, respectively. As shown in Figure 27, in the time interval t∈[160,180]s, the set of visible aircraft ${\bar{Q}}_{1}$ was often empty, leaving the vehicle to fly and overcome obstacles without any swarm rule.

**Figure 29:**
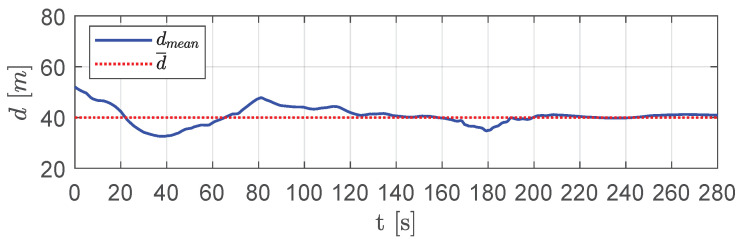
Scenario #3—10 aircraft: mean distance between visible aircraft (blue solid line) compared with desired distance (red dotted line).

Finally, the UAVs re-estabilished the formation in absence of further obstacles. Figure 29 shows the average distance between visible aircraft. There was a clear tendency of it to become equal to the desired distance $\bar{d}$.

During the validation test campaign, the proposed algorithm showed its properties as expected and discussed in the introduction. In the absence of obstacles, aircraft were able to fly together without creating a particular shape of the formation but maintaining mutual distances equal to the desired one. In presence of obstacles, such behavior resulted in a compact formation that split only near the obstacles to avoid collisions.

The absence of a fixed structure and a fixed leader or virtual leader makes the procedure really decentralized, lowering typical implementation and management difficulties [37,91]. During the flight, vehicles can freely change the topology of the swarm, creating sub-swarms as needed and re-composing the overall formation when possible.

It is worth noting that the use of the Delaunay triangulation makes the swarm cooperatively solve a particular packing problem [92] without a fixed external boundary. The objective of this packing problem is to find the shape of the swarm that can be exactly divided in equilateral triangles and whose vertices represent the vehicles positions. The result tended to a hexagonal packing that is the most efficient in terms of occupied area [93].

**Figure 30:**
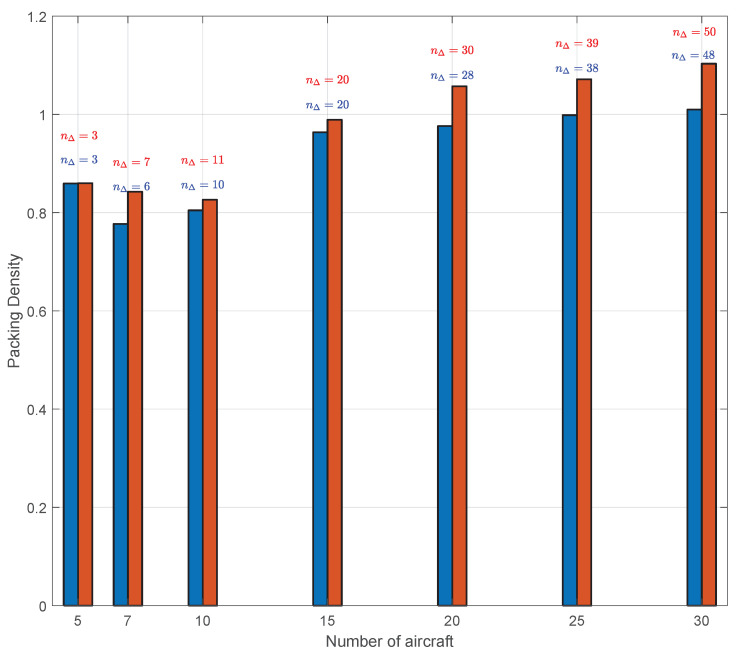
Minimum (blue bars) and maximum swarm packing densities (red bars) in the absence of obstacles. $n_{\Delta }$ describes the number of triangles in a DTG. The values close to 1 highlight the efficiency of the Delaunay-based swarm guidance strategy.

The swarm packing was tested in the absence of obstacles in order to show the packing density, which is defined as follows:

(31) $$density=\frac{N\pi {\bar{d}}^{2}}{4A_{c}}$$

where $A_{c}$ is the area of the region containing all the circles with a diameter equal to the desired distance. Figure 30 shows that the packing density was almost constant also in the presence of a swarm with a large number of vehicles and tended to be approximately equal to 1. Although in a packing problem the hexagonal packing density represents a maximum, here the circles can be overlapped, i.e., vehicles may have mutual distances shorter than the desired one. For this reason, the density reached values around 1.

**Figure 31:**
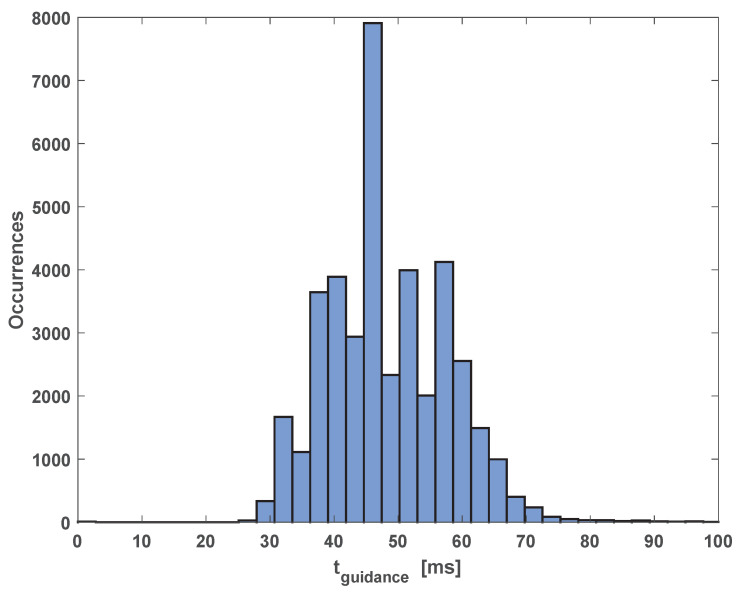
Occurrences of the MPC computational time for the proposed simulations.

Another important feature is that the proposed approach involves a reasonably low computational burden. As shown in Figure 31, the MPC, which is the most demanding part of the algorithm from a computational point of view, never required more than 100 ms on an Intel i7-based laptop for the proposed numerical simulations. This result suggests the possible implementation of the algorithm even on slower CPU architectures, lowering the guidance algorithm sampling frequency.

## 7. Conclusions

In this paper, a decentralized guidance system for swarms of aircraft is proposed. It is based on both a Delaunay triangulation topology, to realize a regular mesh of the formation, and a potential field technique to compute the desired position for each aircraft. The tracking of the desired positions and collision avoidance is then based on a receding horizon model predictive control to optimize the overall performance of the swarm. Vehicles need only to exchange information about position, speed and heading, whereas their on-board control algorithms run independently. Since the fleet formation is not pre-assigned, in the presence of obstacles the swarm can split into several independent sub-swarms to avoid collisions. However, far from the obstacles, the proposed approach allows the vehicles to re-join and re-establish the flight formation. The vehicles tend to self-organize the swarm in order to assume a regular triangle-based formation.

Numerical results, involving different scenarios and fleet configurations, showed the effectiveness of the guidance and control system and underlined several features of the proposed technique.

In particular, the algorithm makes the UAVs formation easily adaptable to different operational environments by changing the swarm configuration and, consequently, the DTG connectivity over time.

This feature is difficult to obtain using an assigned structure or virtual structure as in a leader–follower paradigm. Furthermore, using our approach, the final configuration represents the solution to an optimization problem similar to the packing problem. The use of triangles and, in particular, Delaunay triangulation, gives the swarm the ability to increase the efficiency in terms of packing density. Finally, in terms of future applications, the relatively low computational burden would allow an implementation of the proposed approach also on an embedded low-cost control board. 

## Figures and Tables

**Table 1:** Simulation parameters.

Description	Value
Cruise speed $V_{c}$ [m/s]	5
Minimum speed $V_{min}$ [m/s]	3
Maximum speed $V_{max}$ [m/s]	10
Minimum normal acceleration $a_{min}^{⊥}$ [m/s${}^{2}$]	−5
Maximum normal acceleration $a_{max}^{⊥}$ [m/s${}^{2}$]	5
Minimum tangential acceleration $a_{min}^{\|}$ [m/s${}^{2}$]	−10
Maximum tangential acceleration $a_{min}^{\|}$ [m/s${}^{2}$]	10
Safety distance $d_{safety}$ [m]	15
Aircraft size ${\bar{r}}_{j}\;\forall j=1,\ldots ,N$ [m]	5
Desired distance $\bar{d}$ [m]	40
Weight matrix for tracking error Q	diag([10 10 10 10])
Weight matrix for control effort R	diag([1 1])
Number of steps of the prediction horizon $n_{p}$	10
Number of steps of the control horizon $n_{c}$	5
